# Plasmin Regulation through Allosteric, Sulfated, Small Molecules

**DOI:** 10.3390/molecules20010608

**Published:** 2015-01-05

**Authors:** Rami A. Al-Horani, Rajesh Karuturi, Domonique T. White, Umesh R. Desai

**Affiliations:** Department of Medicinal Chemistry and Institute for Structural Biology and Drug Discovery, Virginia Commonwealth University, Richmond, VA 23219, USA; E-Mails: rkaruturi@vcu.edu (R.K.); d.t.white17696@spartans.nsu.edu (D.T.W.); urdesai@vcu.edu (U.R.D.)

**Keywords:** allosteric inhibitors, library screening, plasmin, sulfated glycosaminoglycan mimetics

## Abstract

Plasmin, a key serine protease, plays a major role in clot lysis and extracellular matrix remodeling. Heparin, a natural polydisperse sulfated glycosaminoglycan, is known to allosterically modulate plasmin activity. No small allosteric inhibitor of plasmin has been discovered to date. We screened an *in-house* library of 55 sulfated, small glycosaminoglycan mimetics based on nine distinct scaffolds and varying number and positions of sulfate groups to discover several promising hits. Of these, a pentasulfated flavonoid-quinazolinone dimer **32** was found to be the most potent sulfated small inhibitor of plasmin (*IC_50_* = 45 μM, efficacy = 100%). Michaelis-Menten kinetic studies revealed an allosteric inhibition of plasmin by these inhibitors. Studies also indicated that the most potent inhibitors are selective for plasmin over thrombin and factor Xa, two serine proteases in coagulation cascade. Interestingly, different inhibitors exhibited different levels of efficacy (40%–100%), an observation alluding to the unique advantage offered by an allosteric process. Overall, our work presents the first small, synthetic allosteric plasmin inhibitors for further rational design.

## 1. Introduction

Plasmin, a trypsin-like serine protease, promotes intravascular dissolution of fibrin clots [1,2]. Plasmin inhibitors are clinically used as antifibrinolytics so as to reduce excessive blood loss during major surgeries such as cardiac surgery with cardiopulmonary bypass [3,4]. Plasmin inhibitors are also used to manage cases of hemophilia, menorrhagia, von Willebrand syndrome, disseminated intravascular coagulation, and thrombolytics-induced bleeding. Plasmin can also be produced at the cell surface as to contribute to the degradation of extracellular matrix resulting in modulation of tissue remodeling, cell invasion, and/or metastasis and chemotaxis. This implies that inhibitors of plasmin could potentially benefit many other pathologies too including angioedema, chronic inflammatory responses, and lymphoid malignancies [1,2].

Nevertheless, only two indirect inhibitors of plasmin are approved for use as antifibrinolytics in the clinic today. Tranexamic acid and ε-aminocaproic acid, two analogs of lysine, bind to the lysine-binding sites in the kringle domains of plasminogen resulting in attenuation of plasmin formation [1]. Both lysine analogs have no direct inhibitory effect on active plasmin leading to their limited efficacy. A direct plasmin inhibitor, which is approved for use in only few countries, is aprotinin. Although useful, aprotinin, a Kunitz-type plasmin inhibitor, was withdrawn from the clinic in the U.S. due to its high risk of mortality and morbidity [5,6]. The two lysine analogs also suffer from significant adverse effects arising from their effects on the central GABA receptor, which provokes convulsive seizures [7]. In particular, the low efficacy of tranexamic acid necessitates administration of high doses (1 to 20 g), which invokes postoperative convulsive seizures, chest tube drainage, and renal dysfunction [8]. Thus, discovering more potent and safer plasmin inhibitors is important and urgent.

Several small molecules are being developed as inhibitors of human plasmin [1]. Each of these is an active site inhibitor, which is typically easier to discover considering the availability of known substrate sequence specificity. Yet, ensuring selective inhibition of plasmin by active site inhibitors is challenging because of the substantial homology to other trypsin-like serine proteases including thrombin, factor Xa, factor XIa, kallikrein, and activated protein C [9]. We reasoned that discovering allosteric inhibitors of plasmin would be advantageous because of the possibility of higher specificity and regulation characteristics.

**Figure 1:**
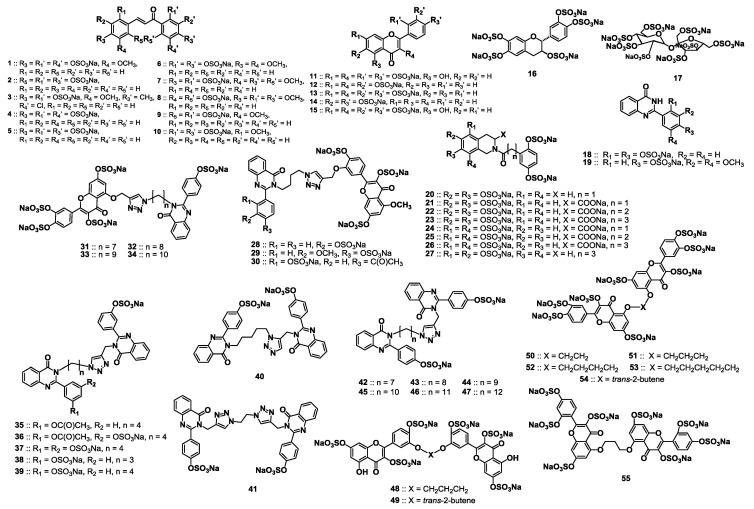
Structures of the sulfated small molecules screened for human plasmin inhibition. The library includes 55 molecules belonging to diverse chemical classes of chalcones (compounds **1**–**10**), flavonoids (**11**–**16**), sucrose octasulfate (**17**), quinazolinones (**18** and **19**), tetrahydroisoquinolines (**20**–**27**), flavonoid-quinazolinone heterodimers (**28**–**34**), bis-quinazolinone homodimers (**35**–**47**), and bis-flavonoid homodimers (**48**–**55**). The sulfated molecules also differed in the number of sulfate groups (1–8/molecule) as well as their spatial orientation.

To realize this, we studied an *in-house* library of 55 sulfated small molecules (Figure 1) considering their similarity to sulfated glycosaminoglycans (GAGs), which had earlier been known to allosterically inhibit plasmin [1]. The focused library was synthesized and screened against human Lys-plasmin using a chromogenic substrate hydrolysis assay to identify several molecules with reasonable activity. In particular, inhibitor **32** inhibited the proteolytic activity of plasmin with an *IC_50_* of 45 μM and efficacy of 100%. Michaelis-Menten kinetic studies revealed that molecule **32** is an allosteric inhibitor. Interestingly, several inhibitors displayed different levels of efficacy (40%–100%), an observation alluding to the possibility of regulating plasmin activity. Molecule **32** is the first homogeneous and non-polymeric allosteric inhibitor of plasmin and is expected to serve as a unique platform to guide future efforts to design highly potent and selective regulators of plasmin.

## 2. Results and Discussion

### 2.1. Rationale for Screening a Focused Library of Sulfated Small Molecules against Human Plasmin

Many different approaches have been utilized to discover and/or rationally design inhibitors of plasmin. These approaches include substrate-based design of linear and cyclic peptidomimetics [10,11,12,13,14], mutagenesis of key residues to engineer Kunitz- and Kazal-type protein/peptide inhibitors [15,16,17], covalent inhibition through a reactive nitrile or aldehyde warhead [18,19], and structure-based computational inhibitor design [20,21]. Each of these approaches typically targets the enzyme’s active site. Yet, the literature supports the idea of allosteric modulation of plasmin’s catalytic activity. For example, heparin is known to bind directly to plasmin with a *K_D_* of 10 nM and induce a conformational change in its active site by interacting with an allosteric site [22,23,24,25]. Likewise, *N*-oleoyl heparin was also found to inhibit plasmin (*IC_50_* = 16 nM) and Lineweaver–Burk analysis indicated noncompetitive inhibition mechanism [26]. In addition, another group of highly sulfated GAG mimetics, e.g., sulfated low molecular weight lignins (CDSO3 *IC_50_* 0.24 μM) [27], chemically modified dextran sulfate derivatives (RG1192 *IC_50_* 2 nM) [28], and sulfated polyvinylalcohol-acrylate copolymers (*IC_50_* 100 nM) [29] have also been reported to inhibit plasmin. 

Sulfated GAGs or sulfated polymeric GAG mimetics are highly heterogeneous polymers, which limits their further development as drugs. We reasoned that small, synthetic, homogenous, non-saccharide GAG mimetics (NSGMs) may offer an avenue for discovering novel plasmin inhibitors. In fact, Desai and co-workers have developed a sizeable number of NSGMs based on various scaffolds including sulfated flavonoids [30,31,32,33], sulfated benzofurans [34,35], sulfated tetrahydroisoquinolines [36], sulfated quinazolinones [37] and sulfated galloyl glucopyranosides [38,39] as modulators of a range of coagulation proteins. The NSGMs resemble sulfated GAGs in the form of presenting one or more sulfate groups to interact with GAG-binding domains on targeted proteins. Specificity of recognition arises from the three-dimensional orientation of key sulfate group(s), which depends on the type of non-saccharide scaffold. Considering that plasmin is known to possess a heparin-binding site, we predicted that one or more NSGM of the many synthesized in our focused library would inhibit plasmin in an allosteric manner.

### 2.2. Chemical Synthesis of the Library of NSGMs

We studied a library of 55 NSGMs representing nine distinct chemical classes of monomeric and dimeric scaffolds (Figure 1). The monomeric scaffolds included chalcones (compounds **1**–**10**), flavonoids (**11**–**16**) [30,31,32], sucrose octasulfate (**17**) [40], quinazolinones (**18** and **19**) [37], and tetrahydro-isoquinolines (**20**–**27**) [36], whereas the dimeric scaffolds comprised flavonoid-quinazolinone heterodimers (**28**–**34**) [37], bis-quinazolinones homodimers (**35**–**47**) [37], and bis-flavonoid homodimers (**48**–**55**). In addition to the inherent diversity of the scaffolds in this library, NSGMs also differed in the number (1 to 8) and orientation of the sulfate groups. NSGMs **11**–**30**, and **35**–**41** were synthesized as reported earlier [30,31,32,33,34,35,36,37]. 

**Scheme 1:**
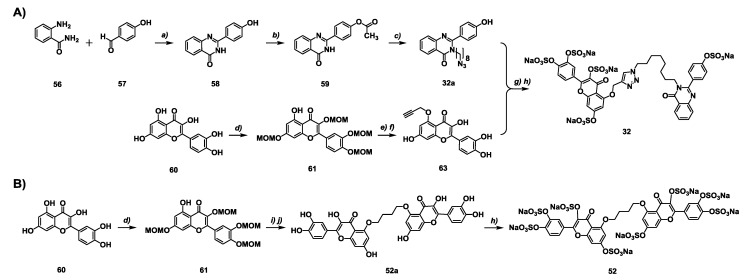
Synthesis of inhibitors **32** (**A**) and **52** (**B**). *a)* NaHSO3, DMA, reflux/overnight, 65%–80%; *b)* Ac_2_O, pyr., DCM, rt, 2 h, 90%; *c)* 1-bromo-n-chloro-octane, K_2_CO_3_, DMF, rt, 12 h, followed by NaN_3_, DMF, overnight 60 °C, 80%–90%; *d)* MOM-Cl, DIPEA, DCM, rt, 12 h; *e)* K_2_CO_3_, propargyl bromide, DMF, rt/2 h, 85%–90%; *f)* 3N HCl, acetone, reflux/overnight, 55%–60%; *g)* CuSO_4_·5H_2_O (1 mol %), sodium ascorbate (5 mol %), DMF/H_2_O (1:1), rt, overnight, 80%–95%; *h)* SO_3_/Me_3_N, TEA, CH_3_CN, microwave, 90 °C, 0.5–6 h, 85%–90%; *i)* K_2_CO_3_, dibromobutane (0.5 equiv), DMF, rt, 6 h, 85%–90%; *j)* *p*-toluenesulfonic acid, MeOH, reflux, 48 h, 55%–65%.

New NSGMs synthesized for the first time included 10 sulfated chalcones (**1**–**10**), six sulfated bis-quinazolinones homodimers (**42**–**47**), four sulfated flavonoid-quinazolinone heterodimers (**31**–**34**), and eight sulfated bis-flavonoid homodimers (**48**–**55**). The synthesis of the new NSGMs is described in detail in Supplementary Information (Schemes S1–S6). Briefly, the synthesis of these NSGMs was achieved in 4 to 8 steps using traditional protection—deprotection chemistry following construction of the base scaffold with appropriate substitution pattern. The final step for the generation of each NSGM was chemical sulfation using trimethylamine-sulfur trioxide at elevated temperature, as described in our studies earlier [30,31,32,33,34,35,36,37,38,39,40,41,42]. For example, the synthesis of two promising plasmin inhibitors (Scheme 1) involved exploitation of the intramolecularly hydrogen bonded 5-OH group of quercetin **60** to selectively introduce a reactive handle (propargyl group in **63** or *in situ* alkyl bromide) that can be coupled with either an alkyl azide containing quinazolinone unit **32a** or MOM-protected quercetin **61** to eventually give inhibitors **32** and **52**, respectively. Overall, the new NSGMs were obtained in high yields (75%–90%) and their structural identity was confirmed using a combination of ^1^H- and ^13^C-NMR spectroscopy and UPLC-MS (See Supplementary Information).

### 2.3. Inhibition of Human Plasmin by the Library of NSGMs

**Figure 2:**
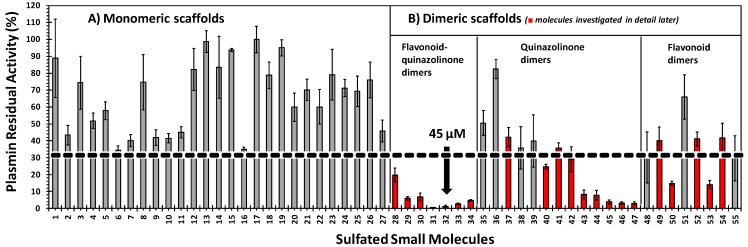
Initial screening of the 55 sulfated small molecules against human plasmin. Screening was performed in 50 mM Tris-HCl buffer at 150 mM NaCl, pH 7.4, and 37 °C using the corresponding chromogenic substrate hydrolysis assay in the presence of 400 μM inhibitor (n ≥ 2). The error bars represents one standard deviation.

The library of synthetic NSGMs was screened for inhibition of human Lys-plasmin using the chromogenic substrate hydrolysis assay, as described earlier [27], under near physiological conditions in Tris-HCl buffer, pH 7.4, containing 150 mM NaCl at 37 °C. Initial screening was performed at 400 μM NSGM concentration and only those agents that displayed ≥70% inhibition were considered for detailed *IC_50_* characterization. As shown in Figure 2, none of the monomeric NSGMs including the highly sulfated disaccharide sucrose octasulfate **17** inhibited plasmin more than 70%. In contrast, several dimeric scaffolds exhibited considerable promise. For example, flavonoid-quinazolinone heterodimers **28**–**34** and flavonoid homodimers **50** and **53** inhibited plasmin more than 90%. Likewise, 7 out of 13 quinazolinone dimers (**40** and **42**–**47**) exhibited plasmin inhibition. Thus, the size and distribution of functional groups appeared to be important to induce plasmin inhibition.

**Figure 3:**
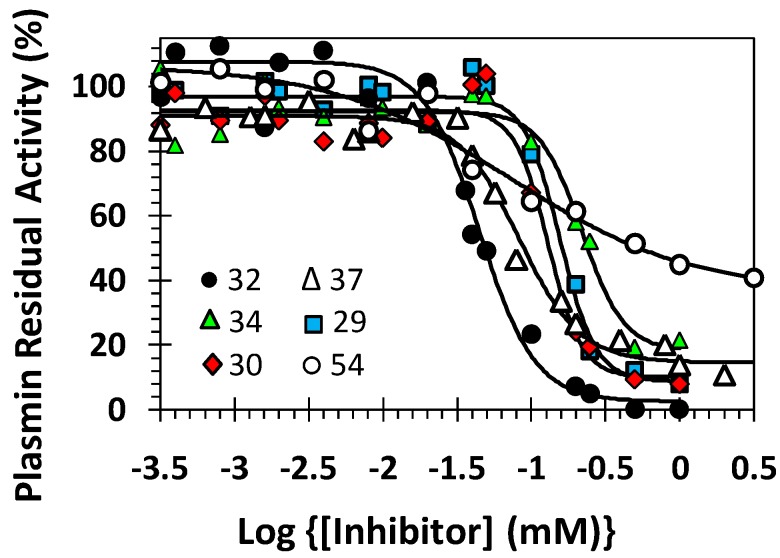
Representative profiles of direct inhibition of human plasmin by sulfated molecules. The inhibition of plasmin was measured spectrophotometrically through a chromogenic substrate hydrolysis assay at pH 7.4 and 37 °C. Solid lines represent sigmoidal fits to the data to obtain *IC_50_*, HS, *Y_M_*, and *Y_O_* using Equation (1), as described in the Experimental Procedures.

To measure the potency and efficacy of these 16 plasmin inhibitors, the sigmoidal dose-dependence of plasmin inhibition was fitted using the logistic equation (See Equation (1) in Experimental Section) to calculate all inhibition parameters (Table 1). Important to emphasize here that the potency of NSGM-based inhibitors was evaluated by measuring their *IC_50_'s* (X-axis), whereas the efficacy was assessed by measuring the overall change in residual plasmin activity (%) (*ΔY* = *Y_M_* − *Y_0_*) (Y-axis). Representative inhibition profiles are shown in Figure 3. Inhibitors **31** and **32** were found to be most potent with *IC_50_* of 56 ± 2 μM and 45 ± 2 μM, respectively, and efficacy of 87% ± 4% and 105% ± 6%, respectively. The two inhibitors are structurally related and belong to the chemical class of flavonoid-quinazolinone hetero-dimers having a quinazolinone moiety substituted with one sulfate group at 4'-position and flavonoid structure with four sulfate groups at positions-3,7,3', and 4'. The two units linked through a tetrazole-containing 11 and 12-atom linker, respectively. The two base scaffolds are connected at *N^3^*-position of quinazolinone and *O^5^*-position of flavonoid.

**Table 1:** Plasmin inhibition by sulfated small molecules. ^a^

Plasmin Inhibitor	*IC_50_* (μM)	HS	ΔY%
**28**	149 ± 5.6 ^b^	3.3 ± 0.7	82 ± 4
**29**	157 ± 5.0	3.6 ± 0.7	88 ± 4
**30**	220 ± 11	3.0 ± 1.3	74 ± 7
**31**	56 ± 2	3.2 ± 0.8	87 ± 4
**32**	45 ± 2	2.3 ± 0.7	105 ± 6
**33**	128 ± 8	1.8 ± 0.4	89 ± 7
**34**	130 ± 6	3.9 ± 1.0	83 ± 5
**35**	642 ± 78	1.4 ± 0.4	88 ± 11
**36**	>1000	ND ^c^	ND
**37**	84 ± 4	2.1 ± 0.4	76 ± 3
**38**	>400	ND	ND
**39**	>400	ND	ND
**40**	239 ± 73	1.1 ± 0.6	87 ±29
**41**	125 ± 9	4.0 ± 1.8	54 ± 5
**42**	161 ± 24	1.0 ± 0.2	92 ± 4
**43**	183 ± 43	1.0 ± 0.3	94 ± 21
**44**	137 ± 11	1.0 ± 0.2	102 ± 9
**45**	98 ± 9	1.7 ± 0.6	94 ± 6
**46**	111 ± 7	2.4 ± 0.4	95 ± 2
**47**	89 ± 7	1.9 ± 0.6	98 ± 7
**48**	621 ± 185	1.3 ± 0.4	89 ± 36
**49**	277 ± 61	1.8 ± 1.0	75 ± 22
**50**	185 ± 68	1.4 ± 0.2	38 ± 15
**51**	~2830	ND	ND
**52**	76 ± 12	1.0 ± 0.2	72 ± 9
**53**	209 ± 25	0.6 ± 0.1	105 ± 8
**54**	75 ± 25	0.7 ± 0.3	71 ± 18
**55**	>400	ND	ND

Notes: ^a^ *IC_50_*, HS, and ΔY values were obtained following non-linear regression analysis of direct inhibition of human plasmin in pH 7.4 buffer at 37 °C. Inhibition was monitored by spectrophotometric measurement of the residual enzyme activity. See details under Experimental Section. ^b^ Errors represent standard error calculated using global fit of the data. ^c^ ND means not determined.

### 2.4. Structure-Activity Relationship of Plasmin Inhibition

To better understand the optimal structural features required for plasmin inhibition, we measured the inhibition profile for 12 additional sulfated NSGMs from the library. The class of sulfated flavonoid-quinazolinone dimers **28**–**34** was the most potent among the three dimeric classes studied with *IC_50_* values of 45–220 μM (Figure 2 and Table 1). The efficacy of inhibition was also found to be high (>74%). As expected, longer linkers in sulfated dimers, such as the 13-atom and 14-atom linkers in **33** and **34**, respectively, diminished the potency ~3-fold relative to **32**, which has a 12-atom linker. Extended linkers are typically expected to increase the structural flexibility of NSGMs which may thermodynamically disfavor their binding to plasmin. The optimal position of a sulfate group on the quinazolinone unit could be either *meta*- (**28**, *IC_50_* 149 μM) or *para*- (**29**, *IC_50_* 157 μM) but not *ortho*- (**30**, *IC_50_* 220 μM). Replacing only the tetrasulfated flavonoid scaffold in inhibitors **31** and **32** with the para-sulfated quinazolinone moiety in **42** and **43**, respectively, decreased the inhibition potency ~3–4 fold (Table 1). This implies that the heterodimeric scaffolds display superior plasmin inhibitory properties. The impact of other variables, e.g., even shorter linker length, number of sulfate groups on quinazolinone and/or flavonoid moities, and variation in linker attachment position, in the class of heterodimers remains to be tested.

The class of quinazolinone homodimers (**35**–**47**) exhibited highly variable inhibition potency (84 ± 4 μM to >1000 μM) and efficacy (54% ± 5% to 102% ± 9%). The most potent inhibitor in this series was the trisulfated inhibitor **37** (*IC_50_* 84 ± 4 μM; efficacy 76% ± 3%). Inhibitor **37** has an 8-atom linker and one para-sulfate group on one quinazolinone unit and two *meta*-sulfate groups on the other unit. Replacing one of the *meta*-position sulfate groups by an acetoxy group as in **36** or hydrogen atom as in **38** and **39** significantly diminished the inhibitory potency by 125-fold and 5-fold, respectively. This further emphasizes the importance of sulfate group for inhibition of plasmin. Yet, considering the *para,para*-disulfated inhibitors **40**–**47**, changing the linker length from eight to 16 or linker nature from 1,4-triazole- to 1,5-triazole or bis-1,4-triazole had only a marginal impact on the *IC_50_* (89–183 μM).

Finally, the class of flavonoid homodimers (**48**–**55**) demonstrated moderate to very weak potency (*IC_50_* 75–2830 μM) and highly variable efficacy (38% ± 15%–105% ± 8%). The two most potent NSGMs, **52** and **54**, displayed *IC_50_* values of 76 ± 12 μM and 75 ± 25 μM, respectively, and similar efficacy of 72% ± 9% and 71% ± 18%. The two molecules have two identical tetrasulfated flavonoid moieties connected by 4-atom linker, which is saturated in the former and unsaturated in the latter. In this class, it appears that the 4-atom linker is optimal because a 2-atom linker (*i.e.*, **50**) and a 5-atom linker (*i.e.*, **53**) induce loss in potency of 2- and 3-fold, respectively. Likewise, other structural variations in number of sulfate groups (8 *versus* 6 sulfates) or position of attachment (5,5- *versus* 3',3'-) for the two flavonoid moieties linked by identical linkers (*i.e.*, **48**, **51**, and **55**) are detrimental for activity (8-, 37-, and 5-fold).

### 2.5. Mechanism of Plasmin Inhibition by NSGMs **32** and **52**

**Figure 4:**
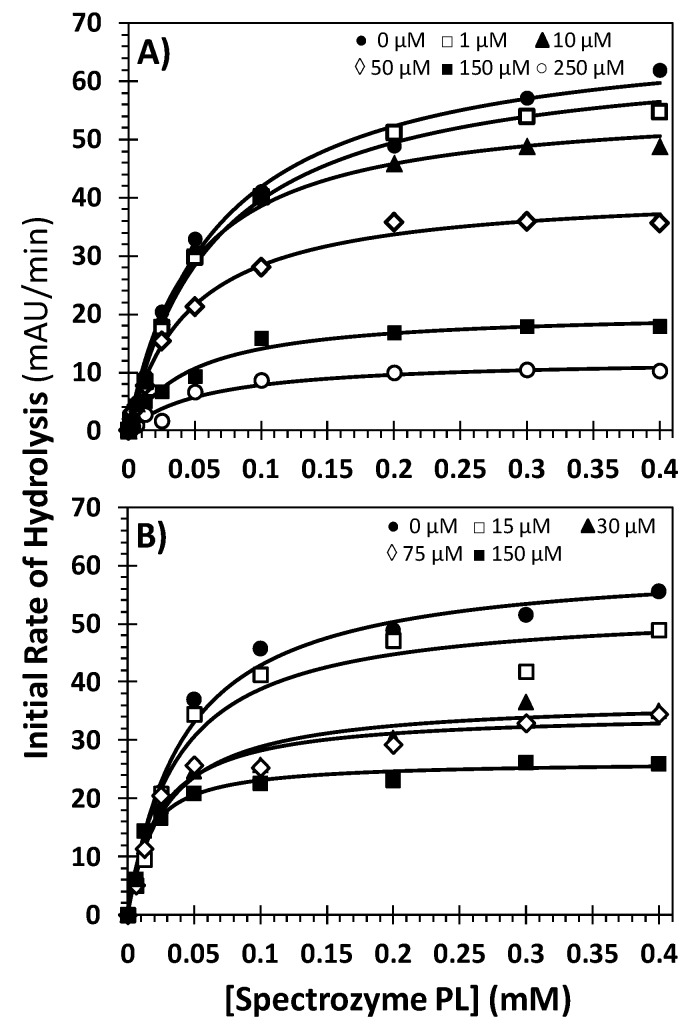
Michaelis-Menten kinetics of chromogenic substrate hydrolysis by human plasmin in the presence of sulfated flavonoid-quinazolinone heterodimer (**32**) (**A**) and flavonoid homodimer (**52**) (**B**). The initial rate of hydrolysis at various substrate concentrations was measured spectrophotometrically in pH 7.4 buffer at 37 °C. Solid lines represent nonlinear regressional fits to the data by the standard Michaelis-Menten equation to yield K_M_ and V_MAX_.

To understand the basis for NSGMs’ plasmin inhibitory potential, the kinetics of Spectrozyme PL, a chromogenic tripeptide substrate, hydrolysis by human plasmin was measured at pH 7.4 and 37 °C in the presence of inhibitors **32** and **52**. As expected, the initial rate of hydrolysis varied in a hyperbolic manner with increasing concentration of Spectrozyme PL at all concentrations of the NSGMs (Figure 4) from which the Michaelis constant (K_M_) and maximal velocity of the reaction (V_MAX_) were calculated (Table 2). The K_M_ for Spectrozyme PL in the absence of **32** was found to be 0.07 ± 0.01 mM, which remained essentially unchanged despite an increase in the inhibitor’s concentration to 250 μM (0.06 ± 0.01 mM). In contrast, the V_MAX_ decreased 5.5-fold from 69.6 ± 3.3 mAU/min to 12.5 ± 1.3 mAU/min for the same range of inhibitor concentration. In the presence of NSGM **52**, both K_M_ and V_MAX_ for Spectrozyme PL decreased approximately 3.5-fold from that in its absence (Table 2). Thus, the two inhibitors (**32** and **52**) appear to differ slightly in their mechanism of plasmin inhibition. While the affinity of the substrate remains unaffected by **32**, it increases in the presence of **52**. Yet, both NSGMs bring about a decrease in the rate of plasmin activity, which is the basis for their functional effect. Technically, the mechanism induced by **32** is called noncompetitive inhibition, while that induced by **52** is called uncompetitive inhibition. While uncompetitive inhibitor **52** requires that a plasmin-substrate Michaelis complex must be formed, noncompetitive inhibitor **32** can occur with or without the substrate present. In either case, the NSGMs appear to bring about changes in the active site of plasmin that induces dysfunction in its catalytic apparatus. Both mechanisms arise from allosteric binding of NSGMs **32** and **52** to plasmin. Thus, the NSGMs studied in this work parallel the growing class of allosteric inhibitors of heparin-binding enzymes reported in the literature to date, such as low molecular weight lignins [27], sulfated benzofurans [34,35], sulfated tetrahydroisoquinolines [36], sulfated quinazolinones [37], and sulfated pentagalloyl glucopyranoses [38,39].

**Table 2:** Hydrolysis of Spectrozyme PL by plasmin in presence of NSGMs **32** and **52**. ^a^

Inhibitor	Conc. (μM)	K_M_ (mM)	V_MAX_ (mAU/min)
**32**	0	0.07 ± 0.01 ^b^	69.6 ± 3.3
	1	0.07 ± 0.01	65.8 ± 2.1
	10	0.05 ± 0.01	56.4 ± 2.3
	50	0.05 ± 0.01	41.4 ± 1.6
	150	0.05 ± 0.01	20.7 ± 1.1
	250	0.06 ± 0.01	12.5 ± 1.3
**52**	0	0.04 ± 0.01	61.0 ± 2.4
	15	0.04 ± 0.01	52.9 ± 2.9
	30	0.03 ± 0.01	37.0 ± 1.5
	75	0.023 ± 0.004	34.8 ± 1.5
	150	0.014 ± 0.002	26.4 ± 0.8

Notes: ^a^ K_M_ and V_MAX_ values of plasmin chromogenic substrate hydrolysis by human plasmin were measured as described in the Experimental Section. mAU indicates milliabsorbance units. ^b^ Error represents ± 1 S.E.

### 2.6. Selectivity Studies: Direct Inhibition of Thrombin and Factor Xa of the Coagulation Cascade

The rationale behind targeting an allosteric site on plasmin was to achieve inhibition specificity over the closely related serine proteases. In contrast to active sites, allosteric sites are not as highly conserved and therefore present a powerful opportunity for discovering a selective modulator of enzyme function. To assess the specificity features of inhibitors **31**, **32**, **52**, and **54**, two closely related coagulation enzymes, thrombin and factor Xa, were studied. Using appropriate small peptide-based chromogenic substrates, the fractional residual thrombin and factor Xa activities were measured under the physiological condition of pH 7.4 and 37 °C. The *IC_50_* values of these inhibitors against thrombin were > 500 μM suggesting a selectivity index of at least 7–10-fold, whereas the *IC_50_* values of same inhibitors against factor Xa were > 250 μM suggesting a selectivity index of about 5-fold. Thus, allosteric inhibitors of plasmin identified in this study are selective.

## 3. Experimental Section

### 3.1. Chemicals, Reagents, Analytical Chemistry, Enzymes, Peptides

Anhydrous CH_2_Cl_2_, THF, CH_3_CN, DMF, DMA and acetone were purchased from Sigma-Aldrich (Milwaukee, WI, USA) or Fisher (Pittsburgh, PA, USA) and used as such. Other solvents used were of reagent gradient and used as purchased. Analytical TLC was performed using UNIPLATE^TM^ silica gel GHLF 250 um pre-coated plates (Analtech, Newark, DE, USA). Column chromatography was performed using silica gel (200–400 mesh, 60 Å) from Sigma-Aldrich. Chemical reactions sensitive to air or moisture were carried out under nitrogen atmosphere in oven-dried glassware. Reagent solutions, unless otherwise noted, were handled under a nitrogen atmosphere using syringe techniques. Flash chromatography was performed using Teledyne ISCO (Lincoln, NE, USA) Combiflash RF system and disposable normal silica cartridges of 30–50 µ particle size, 230–400 mesh size and 60 Å pore size. The flow rate of the mobile phase was in the range of 18 to 35 mL/min and mobile phase gradients of ethyl acetate/hexanes and CH_2_Cl_2_/CH_3_OH were used to elute compounds. Human plasmin was obtained from Haematologic Technologies (Essex Junction, VT, USA). Stock solutions of plasmin and thrombin were prepared in 50 mM Tris-HCl buffer, pH 7.4, containing 150 mM NaCl, 0.1% PEG8000, and 0.02% Tween80. Stock solution of factor Xa was prepared in 20 mM Tris-HCl buffer, pH 7.4, containing 100 mM NaCl, 2.5 mM CaCl_2_, 0.1% PEG8000, and 0.02% Tween80. Plasmin (Spectrozyme PL), thrombin (Spectrozyme TH), and factor Xa (Spectrozyme FXa) chromogenic substrates were all obtained from American Diagnostica (Greenwich, CT, USA).

### 3.2. Chemical Characterization of Compounds

^1^H- and ^13^C-NMR were recorded on a Bruker-400 MHz spectrometer in either CDCl_3_, CD_3_OD, acetone-*d*_6_, DMSO-*d*_6_, or D_2_O. Signals, in part per million (ppm), are either relative to the internal standard or to the residual peak of the solvent. The NMR data are reported as chemical shift (ppm), multiplicity of signal (s = singlet, d = doublet, t = triplet, q = quartet, dd = doublet of doublet, m = multiplet), coupling constants (Hz), and integration. ESI-MS of compounds were recorded using Waters Acquity TQD MS spectrometer in positive or negative ion mode. Samples were dissolved in methanol and infused at a rate of 20 μL/min. For HRMS measurements, a Perkin Elmer AxION 2 TOF MS was used in negative ion mode. Ionization conditions on both instruments were optimized for each compound to maximize the ionization of the parent ion. Generally, the extractor voltage was set to 3 V, the Rf lens voltage was 0.1 V, the source block temperature was set to 150 °C, and the desolvation temperature was about 250 °C. The purity of each final compound was greater than 95% as determined by UPLC-MS. We report here the characterization data for inhibitors **31**, **32**, **52**, and **54**. The characterization data of all inhibitors and their intermediates are reported in the Supplementary Information.

*3-(8-(4-((2-(3,4-O,O-Disulfonato-phenyl)-3,7-O,O-disulfonato-4-oxo-4H-chromen-5-yloxy)methyl)-1H-1,2,3-triazol-1-yl)heptyl)-2-(4-O-sulfonato-phenyl)quinazolin-4(3H)-one, Pentasodium salt* (**31**). ^1^H-NMR (DMSO-*d*_6_): 8.42 (d, *J* = 8.7 Hz, 2 H), 8.31–8.28 (m, 1 H), 8.15–8.11 (m, 3 H), 7.93–7.9 (m, 2 H), 7.65–7.62 (m, 2 H), 7.34 (d, *J* = 8.7 Hz, 2 H), 7.13–7.07 (m, 1 H), 6.85–6.81 (m, 1 H), 5.24 (s, 2 H), 4.71 (t, *J* = 6.4 Hz, 2 H), 4.37 (t, *J* = 7.9 Hz, 2 H), 1.93–1.85 (m, 4 H) 1.55–1.24 (m, 6 H). ^13^C-NMR (D_2_O): 169.04, 160.39, 157.96, 155.86, 154.12, 148.67, 143.76, 135.18, 132.09, 129.78, 128.80, 127.34, 124.34, 123.16, 119.08, 115.13, 114.19, 114.03, 113.96, 66.76, 52.80, 49.42, 29.23, 28.61, 28.35, 25.29, 25.18. MS (ESI) calculated for C_39_H_30_N_5_Na_5_O_24_S_5_ [(M−Na)]^−^, *m/z* 1203.94, found [(M−2Na)]^2−^, *m/z* 590.38.

*3-(8-(4-((2-(3,4-O,O-Disulfonato-phenyl)-3,7-O,O-disulfonato-4-oxo-4H-chromen-5-yloxy)methyl)-1H-1,2,3-triazol-1-yl)octyl)-2-(4-O-sulfonato-phenyl)quinazolin-4(3H)-one, Pentasodium salt* (**32**). ^1^H-NMR (DMSO-*d*_6_): 8.44 (d, *J* = 8.8 Hz, 2 H), 8.32–8.27 (m, 1 H), 8.1–8.12 (m, 3 H), 7.9–7.92 (m, 2 H), 7.6–7.61 (m, 2 H), 7.34 (d, *J* = 8.8 Hz, 2 H), 7.1–7.07 (m, 1 H), 6.8–6.82 (m, 1 H), 5.23 (s, 2 H), 4.71 (t, *J* = 6.4 Hz, 2 H), 4.38 (t, *J* = 7.9 Hz, 2 H), 1.9–1.85 (m, 4 H) 1.5–1.23 (m, 8 H). ^13^C-NMR (D_2_O): 169.03, 160.23, 158.86, 156.07, 151.06, 148.58, 142.20, 134.15, 131.82, 129.96, 128.96, 127.30, 126.79, 124.30, 123.21, 119.83, 115.34, 114.37, 114.17, 66.81, 52.81, 49.40, 29.70, 28.54, 28.27, 28.12, 25.78, 25.44. MS (ESI) calculated for C_40_H_32_N_5_Na_5_O_24_S_5_ [(M−Na)]^−^, *m/z* 1217.95, found [(M−2Na)]^2−^, *m/z* 597.39.

*5,5'-(Butane-1,4-diylbis(oxy))bis(2-(3,4-O,O-disulfonato-phenyl)-3,7-O,O-disulfonato-4H-chromen-4-one), Octasodium salt* (**52**). ^1^H-NMR (DMSO-*d_6_*): 8.15–8.07 (m, 4 H), 7.64 (d, *J* = 9 Hz, 2 H), 7.04 (d, *J* = 1.8 Hz, 2 H), 6.72 (s, 2 H), 4.15 (s, 4 H), 2.11 (s, 4H). ^13^C-NMR (DMSO-*d*_6_): 173.0, 159.41, 158.31, 157.01, 153.27, 146.41, 142.92, 135.30, 124.50, 123.59, 119.80, 118.77, 109.42, 100.02, 98.85, 68.81, 25.5. ESI-MS calculated for C_34_H_18_Na_8_O_38_S_8_ [(M+Na)]^+^, *m/z* 1497.95, found [(M−8Na+8HxA)+2HxA]^2+^, *m/z* 1156.367.

*(E)-5,5'-(But-2-ene-1,4-diylbis(oxy))bis(2-(3,4-O,O-disulfonato-phenyl)-3,7-O,O-disulfonato-4H-chromen-4-one), Octasodium salt* (**54**). ^1^H-NMR (DMSO-*d*_6_): 8.07 (d, *J* = 2.2 Hz, 2 H), 8.0 (d, *J* = 6.7 Hz, 2 H), 6.98 (d, *J* = 1.9 Hz, 2 H), 6.63 (d, *J* = 1.9 Hz, 2 H), 6.39 (s, 2 H), 4.64 (s, 4 H). ^13^C-NMR (DMSO-*d*_6_): 173.24, 158.43, 158.25, 157.08, 154.28, 148.43, 143.82, 134.31, 123.50, 123.28, 119.84, 118.78, 108.45, 100.05, 98.87, 75.81. ESI-MS calculated for C_34_H_16_Na_8_O_38_S_8_ [(M+Na)]^+^, *m/z* 1495.91, found [(M−8Na+8HxA)+2HxA]^2+^, *m/z* 1155.620.

### 3.3. General Procedure of Chemical Sulfation of Small Molecules

Sulfation of polyphenolic precursors was achieved using microwave assisted chemical sulfation as described earlier [30,31,32,33,34,35,36,37,38,39,40,41,42]. Briefly, to a stirred solution of polyphenol in anhydrous CH_3_CN (1–5 mL) at room temperature, Et_3_N (10 equivalents per -OH group) and Me_3_N:SO_3_ complex (6 equivalents per -OH) was added. The reaction vessel was sealed and microwaved (CEM Discover, Cary, NC, USA) for 0.5–8 h at 90–100 °C. The reaction mixture was cooled and transferred to a round bottom flask and volume reduced as much as possible under low pressure conditions at 25 °C. The reaction mixture was then directly loaded on to a flash chromatography column and purified using dichloromethane and methanol solvent system (5%–20%) to obtain the sulfated molecules. The samples were concentrated and reloaded onto a SP Sephadex C-25 column for sodium exchange. Appropriate fractions were pooled, concentrated *in vacuo*, and lyophilized to obtain a white powder. The reaction time was optimized depending on the scaffold and it ranged from 30 min to 8 h at 90–100 °C. All microwave-assisted sulfation reactions were quantitative with a minimum yield of 75%.

### 3.4. Direct Inhibition of Human Plasmin by Sulfated Small Molecules

Direct inhibition of human Lys-plasmin was measured using a chromogenic substrate hydrolysis assay on a microplate reader (FlexStation III, Molecular Devices, Sunnyvale, CA, USA), as reported earlier [27]. Briefly, to each well of a 96-well microplate containing 85 µL of 50 mM Tris-HCl buffer, pH 7.4, containing 150 mM NaCl, 0.1% PEG8000, and 0.02% Tween80 at 37 °C was added 5 µL potential NSGM-based inhibitor (20–100 mM aqueous solution) (or vehicle alone) and 5 µL enzyme. The final concentration of the enzyme was 20 nM. After 5 min incubation, 5 µL of 1 mM Spectrozyme PL was rapidly added and the residual enzyme activity was measured from the initial rate of increase in A_405_. Relative residual enzyme activity (*Y*) as a function of the concentration of sulfated molecule was fitted using logistic equation 1 to obtain the potency (*IC_50_*), efficacy (*ΔY*) and Hill slope (*HS*) of inhibition. In this equation, *Y*_M_ and *Y*_0_ are the maximal and minimal values of *Y*:

(1) $$Y\;=\;Y_{0}+\frac{Y_{M}-Y_{0}}{1+10^{\left(log{\left[Inhibitor\right]}_{0}\;-\;logIC_{50}\right)\;\times HS}}$$

In this equation, *Y* is the ratio of residual plasmin activity in the presence of inhibitor to that in its absence (fractional residual activity). *Y_M_* and *Y_0_* are the maximum and minimum possible values of the fractional residual proteinase activity. The difference between these two values is used to evaluate the inhibitor’s efficacy to reduce the residual enzyme activity under the assay condition. *IC_50_* is the concentration of the inhibitor that results in 50% inhibition of enzyme activity and it is used to evaluate its potency. 

### 3.5. Michaelis-Menten Kinetics of Spectrozyme PL Hydrolysis by Plasmin in the Presence of Molecules **32** and **52**

The initial rate of Spectrozyme PL hydrolysis by human plasmin (20 nM) was monitored from the linear increase in absorbance at 405 nm corresponding to less than 10% consumption of the substrate. The initial rate was measured as a function of various concentrations of the substrate (0–400 μM) in the presence of fixed concentration of inhibitor (**32**) (0–250 μM) or inhibitor (**52**) (0–150 μM) in 50 mM Tris-HCl buffer, pH 7.4, 150 mM NaCl at 37 °C. The data were fitted by Michaelis-Menten Equation (2) to determine *K_M,app_* and V_MAX_:

(2) $$V=\;\frac{V_{MAX}\;[S]}{K_{M}+\;[S]}$$

### 3.6. Selectivity Studies: Direct Inhibition of Thrombin and Factor Xa of the Coagulation Cascade

Direct inhibition of thrombin and factor Xa by **31**, **32**, **52**, and **54** was measured using a chromogenic substrate hydrolysis assay on a microplate reader (FlexStation III, Molecular Devices), as reported earlier [43]. Briefly, to each well of a 96-well microplate containing 185 μL of 20–50 mM Tris-HCl buffer, pH 7.4, containing 100–150 mM NaCl, 0.1% PEG8000 and 0.02% Tween80 at either 25 °C (thrombin) or 37 °C (factor Xa) was added 5 μL of 10–20 mM inhibitor (or vehicle) and 5 μL of the enzyme. The final concentrations of the enzymes were 6 nM (thrombin) and 1.09 nM (factor Xa). After 10 min incubation, 5 μL 1.0 mM Spectrozyme TH or 2.5 mM Spectrozyme FXa, was rapidly added and the residual enzyme activity was measured from the initial rate of increase in A405. Relative residual enzyme activity (Y, activity in the presence of inhibitor to that in its absence) as a function of the concentration of inhibitor was measured. 

## 4. Conclusions 

This work reports the first group of small, homogenous molecules that allosterically inhibit human Lys-plasmin. In particular NSGMs **31**, **32**, **52** and **54** are interesting initial discoveries that provide the foundation necessary for further design of advanced molecules. Allosteric inhibition usually conveys two advantages over active site inhibitors. First, allosteric sites are generally less conserved among proteins of the same superfamily. This is expected to provide enhanced specificity of action. Second, allosteric sites in principle may afford variable control over enzyme function (ΔY not necessarily 100%). Such variable control of enzyme activity is critical for plasmin, which is involved in multiple pathophysiological conditions. This work demonstrates this aspect more clearly than any example known in the literature. For example, NSGMs **41** and **50** display efficacies of only about 50% (54% ± 5% and 38% ± 15%, respectively), which implies that even at saturation, these inhibitors induce only half maximal reduction in plasmin activity. Such controlled reduction in plasmin function can help maintain balance between hemostasis and bleeding, possibly resulting in reduced adverse consequences. Also, several NSGMs display efficacies of ~70% (e.g., **30**, **37**, **49**, **52**, and **54**), while others (e.g., **32**, **42**–**47**) display nearly 100% inhibition (Table 1). The only other NSGM-related work described in the literature pertains to the monosulfated benzofuran class of molecules by Sidhu *et al.* that presents one molecule with efficacy of less than 50% [34]. Thus, plasmin appears to present a system to rigorously test the concept of controlled allosteric regulation as an avenue for avoiding adverse consequences.

A significant number of sulfated NSGMs being reported here are new and have not been studied earlier. The NSGMs display moderate potency with *IC_50_* values of 45–75 μM and represent promising “hits” that can be expected to guide future efforts. Despite the moderate potency, the promise of advancing the field of selective allosteric inhibitors of plasmin is good because of prior work in the area of allosteric inhibitors of thrombin, a related coagulation enzyme [34,35,44]. In this case, a rational design process transformed the initial moderate activity to sub-micromolar activity in few steps. In fact, we predict that coupling one more monomers to these initial dimeric “hits” will improve the inhibition profile greatly. Additionally, the NSGMs discovered here may also serve as chemical biology tools to help understand biochemical and pharmacological facets of plasmin function. 

Tranexamic acid and ɛ-aminocaproic acid are the only plasmin modulators in the clinical use. Despite their success in reducing blood loss associated with major surgeries, the two antifibrinolytics suffer from severe lack of efficacy [1,3,4]. Specificity is also a concern as these lysine analogs may recognize a negatively charged domain(s) on non-target protein(s). An example of this is seizure attacks in some patients. Aprotinin, a plasmin inhibitor in limited use in some countries, also suffers from a lack of specificity [1,5,6]. Several plasmin inhibitors being developed as antifibrinolytics include KDI-L17R [15] textilinin-1 [16] and cyclic peptidomimetics [13,14]. Yet, these are active site inhibitors and their selectivity for plasmin is challenging because of the similarity of active site geometries of a number of trypsin-like proteases. Allosteric inhibitors being presented in this work should provide a fundamentally new route to plasmin inhibition. Toward this end, a putative binding site for NSGMs on the catalytic domain of plasmin was earlier proposed using a computational approach. The putative heparin-binding site includes Arg637, Arg644, Lys645, Lys651, Arg776, and Arg779 in addition to other hydrophobic amino acids. Specifically, this binding site was claimed to be a plausible binding site for the highly sulfated low molecular weight lignins [27], and hence, it could be exploited in rational design of more potent allosteric inhibitors of plasmin.

Overall, the discovery of inhibitors **31**, **32**, **52** and **54** presents a proof-of-concept that homogeneous sulfated NSGMs afford allosteric inhibition of human Lys-plasmin. These small sulfated NSGMs offer many advantages including i) adequate aqueous solubility (>25 mg/mL), which is expected to help antifibrinolytic use during surgeries, ii) limited cellular and central nervous system toxicity arising from their highly charged nature, iii) reasonable chemical stability, particularly under neutral and basic conditions [45], and iv) ease of chemical synthesis [41,46,47]. Further efforts are necessary to develop these sulfated NSGMs into clinically relevant molecules.

## Supplementary Materials

Experimental parts including synthetic and identification protocols, synthetic schemes, and characterization data (^1^H- and ^13^C-NMR, and MS/ESI) of new molecules are provided in supplementary information. Details of procedures and equations of inhibition studies as well as Michaelis-Menten kinetics are also provided. This material is available free of charge via the online version of the paper. Supplementary materials can be accessed at: http://www.mdpi.com/1420-3049/20/01/0608/s1.
