# Supplementary material for: Plasmin Regulation through Allosteric, Sulfated, Small Molecules
Source: Molecules. 2015 Jan 5;20(1):608–24. doi: 10.3390/molecules20010608 (PMC6272155; doi:10.3390/molecules20010608)
Supplement: Supplementary file 1 [file molecules-20-00608-s001.pdf]

Supplementary Materials

Table of Contents	Page
**1. Experimental Procedures**	S2–S3
Chemical, reagents, analytical chemistry, enzymes, and peptides.	S2
Chemical characterization of compounds.	S2
General procedure of chemical sulfation of small molecules.	S2
Direct inhibition of human plasmin by sulfated small molecules.	S3
Michaelis-Menten kinetics of Spectrozyme PL hydrolysis by plasmin in the presence of inhibitor 32 or 52.	S3
**2. Synthesis and Spectral Data for the Newly Synthesized Sulfated Molecules**	S3–S19
Chemical sulfation and spectral data of chalcones. (**1**–**10**) (Scheme 1)	S3–S5
Chemical synthesis and spectral data of flavonoid-quinazolinone *hetero*-dimers. (**31**–**34**) (Scheme 2)	S5–S9
Chemical synthesis and spectral data of sulfated quinazolinone *homo*-dimers. (**42**–**47**) (Scheme 3)	S9–S12
Chemical synthesis and spectral data of sulfated flavonoid *homo*-dimers. (**48**–**55**) (Schemes 4–6)	S13–S19
**3. References**	S19

1. Experimental Procedures

Chemicals, reagents, analytical chemistry, enzymes, peptides. Anhydrous CH_2Cl_2, THF, CH_3CN, DMF, DMA and acetone were purchased from Sigma-Aldrich (Milwaukee, WI) or Fisher (Pittsburgh, PA) and used as such. Other solvents used were of reagent gradient and used as purchased. Analytical TLC was performed using UNIPLATE^{TM} silica gel GHLF 250 um pre-coated plates (ANALTECH, Newark, DE). Column chromatography was performed using silica gel (200–400 mesh, 60 Å) from Sigma-Aldrich. Chemical reactions sensitive to air or moisture were carried out under nitrogen atmosphere in oven-dried glassware. Reagent solutions, unless otherwise noted, were handled under a nitrogen atmosphere using syringe techniques. Flash chromatography was performed using Teledyne ISCO (Lincoln, NE) Combiflash RF system and disposable normal silica cartridges of 30–50 μ particle size, 230–400 mesh size and 60 Å pore size. The flow rate of the mobile phase was in the range of 18 to 35 mL/min and mobile phase gradients of ethyl acetate/hexanes and CH_2Cl_2/CH_3OH were used to elute compounds. Human plasmin was obtained from Haematologic Technologies (Essex Junction, VT). Stock solutions of plasmin was prepared in 50 mM Tris-HCl buffer, pH 7.4, containing 150 mM NaCl, 0.1% PEG8000, and 0.02% Tween80. Plasmin chromogenic substrate (Spectrozyme PL) was obtained from American Diagnostica (Greenwich, CT).

Chemical characterization of compounds. ^{1}H- and ^{13}C-NMR were recorded on Bruker-400 MHz spectrometer in either $CDCl_3$, CD_3OD, acetone-*d*$_6$, DMSO-D_6, or D_2O. Signals, in part per million (ppm), are either relative to the internal standard or to the residual peak of the solvent. The-NMR data are reported as chemical shift (ppm), multiplicity of signal (s = singlet, d = doublet, t = triplet, q = quartet, dd = doublet of doublet, m = multiplet), coupling constants (Hz), and integration. ESI-MS of compounds were recorded using Waters Acquity TQD-MS spectrometer in positive or negative ion mode. Samples were dissolved in methanol and infused at a rate of 20 μL/min. For HRMS measurements, a Perkin Elmer AxION 2 TOF MS was used in negative ion mode. Ionization conditions on both instruments were optimized for each compound to maximize the ionization of the parent ion. Generally, the extractor voltage was set to 3 V, the Rf lens voltage was 0.1 V, the source block temperature was set to 150 °C, and the desolvation temperature was about 250 °C. The purity of each final compound was greater than 95% as determined by UPLC-MS.

General procedure of chemical sulfation of small molecules. Sulfation of polyphenolic precursors was achieved using microwave assisted chemical sulfation as described earlier [1]. Briefly, to a stirred solution of polyphenol in anhydrous CH_3CN (1–5 mL) at room temperature, Et_3N (10 equvi per –OH group) and $Me_3N{:}SO_3$ complex (6 equvi per –OH) was added. The reaction vessel was sealed and micro-waved (CEM Discover, Cary, NC, USA) for 30 min–8 h at 90–100 °C. The reaction mixture was cooled and transferred to a round bottom flask and volume reduced as much as possible under low pressure conditions at 25 °C. The reaction mixture was then directly loaded on to a flash chromatography column and purified using dichloromethane and methanol solvent system (5%–20%) to obtain the sulfated molecules. The samples were concentrated and re-loaded onto a SP Sephadex C-25 column for sodium exchange. Appropriate fractions were pooled, concentrated in *vacuo*, and lyophilized to obtain a white powder. The reaction time was optimized depending on the scaffold and it ranged from 30 min to 8 h at 90–100 °C. All microwave-assisted sulfation reactions were quantitative with a minimum yield of 75%.

Direct inhibition of human plasmin by sulfated small molecules. Direct inhibition of plasmin was measured using a chromogenic substrate hydrolysis assay on a microplate reader (FlexStation III, Molecular Devices), as reported earlier [2]. Briefly, to each well of a 96-well microplate containing 85 μL of 50 mM Tris-HCl buffer, pH 7.4, containing 150 mM NaCl, 0.1% PEG8000, and 0.02% Tween80 at 37 °C was added 5 μL potential inhibitor (or vehicle alone) and 5 μL enzyme. The final concentration of the enzyme was 20 nM. After 5 min incubation, 5 μL of 1 mM Spectrozyme PL was rapidly added and the residual enzyme activity was measured from the initial rate of increase in A_{405}. Relative residual enzyme activity (*Y*, activity in the presence of inhibitor to that in its absence) as a function of the concentration of SPGG derivative was fitted using logistic Equation (1) to obtain the potency (IC_{50}), efficacy (ΔY) and Hill slope (*HS*) of inhibition. In this equation, Y_M and Y_0 are the maximal and minimal values of *Y*.

$$Y = Y_0 + \frac{Y_M - Y_0}{1 + 10^{(log[Inhibitor]_0 - logIC_{50}) \times HS}} \quad (1)$$

Michaelis-Menten kinetics of Spectrozyme PL hydrolysis by plasmin in the presence of molecule (32). The initial rate of Spectrozyme PL hydrolysis by human plasmin (20 nM) was monitored from the linear increase in absorbance at 405 nm corresponding to less than 10% consumption of the substrate. The initial rate was measured as a function of various concentrations of the substrate (0–400 μM) in the presence of fixed concentration of inhibitor (**32**) (0–250 μM) or inhibitor (**52**) (0–150 μM) in 50 mM Tris-HCl buffer, pH 7.4, 150 mM NaCl at 37 °C. The data were fitted by Michaelis-Menten Equation (2) to determine $K_{M,app}$ and V_{MAX}.

$$V = \frac{V_{MAX}\ [S]}{K_M + [S]} \quad (2)$$

2. Synthesis and Spectral Data for the Newly Synthesized Sulfated Molecules

Sulfated chalcones **(1–10)**

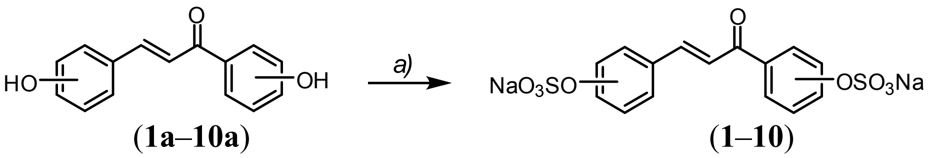

Scheme S1. *a)* SO_3:Me_3N, TEA, CH_3CN, microwave, 30 min, 90 °C, 85%–90%.

(**1**). ^{1}H-NMR (DMSO-d_6, 400 MHz): 7.7 (d, 1 H, *J* = 15.71 Hz), 7.46–7.41 (m, 4 H), 7.32 (d, 1 H, *J* = 2.96 Hz), 7.18 (m, 1 H), 7.15 (d, *J* = 6.7 Hz), 3.73 (s, 3 H). ^{13}C-NMR (DMSO-d_6, 100 MHz): 189.85, 150.40, 149.16, 148.12, 144.82, 141.82, 131.85, 130.14, 125.63, 124.92, 122.43, 122.30, 120.97, 120.12, 111.19, 55.64. ESI-MS calculated for $C_{16}H_{11}Na_3O_{14}S_3$ $[(M+Na)]^+$, *m/z* 615.90, found $[(M-3Na+3\ HxA)+1HxA]^+$, *m/z* 931.65.

(**2**). ^{1}H-NMR (DMSO-d_6, 400 MHz): 7.92–7.78 (m, 3 H), 7.51 (d, 1 H, *J* = 8.68 Hz), 7.41 (d, 1 H, *J* =7.92 Hz), 7.25 (m, 2 H), 7.06–7.02 (m, 2 H). ^{13}C-NMR (DMSO-d_6, 100 MHz): 188.99, 157.32, 153.57, 152.65, 135.94, 130.40, 130.14, 127.35, 126.80, 126.58, 126.42, 123.39, 121.57, 115.11, 113.15.

ESI-MS calculated for $C_{15}H_9Na_3O_{13}S_3$ [(M+Na)]$^+$, *m/z* 585.32, found [(M−3Na+3 HxA)+1HxA]$^+$, *m/z* 901.62.

(**3**). ^{1}H-NMR (DMSO-*d*$_6$, 400 MHz): 7.85 (d, 1 H, *J* = 15.69 Hz), 7.61 (d, 2 H, *J* = 4.72 Hz), 7.55–7.49 (m, 3 H), 7.19 (d, 1 H, *J* = 8.36 Hz), 3.81 (s, 3 H), 2.37 (s, 3 H). ^{13}C-NMR (DMSO-*d*$_6$, 100 MHz): 188.44, 151.22, 150.40, 145.07, 140.10, 130.66, 129.89, 129.14, 127.99, 125.10, 124.48, 122.49, 120.08, 111.12, 55.63. ESI-MS calculated for $C_{17}H_{13}ClNa_2O_{10}S_2$ [(M+Na)]$^+$, *m/z* 545.84, found [(M−2Na+2 HxA)+1HxA]$^+$, *m/z* 782.46.

(**4**). ^{1}H-NMR (DMSO-*d*$_6$, 400 MHz): 7.70 (m, 3 H), 7.51 (m, 2 H), 7.31 (d, 1 H, *J* = 2.96 Hz), 7.23 (m, 3 H). ^{13}C-NMR (DMSO-*d*$_6$, 100 MHz): 190.16, 155.45, 149.17, 148.04, 141.54, 131.98, 129.66, 129.45, 125.51, 124.74, 122.43, 120.90, 120.12. ESI-MS calculated for $C_{15}H_9Na_3O_{13}S_3$ [(M+Na)]$^+$, *m/z* 585.39, found [(M−3Na+3 HxA)+1HxA]$^+$, *m/z* 901.58.

(**5**). ^{1}H-NMR (DMSO-*d*$_6$, 400 MHz): 7.74–7.70 (m, 1 H), 7.60 (d, 2 H, *J* = 8.64 Hz), 7.50–7.43 (m, 2 H), 7.25 (d, 1 H, *J* = 2.24 Hz), 7.13 (d, 2 H, *J* = 8.63 Hz), 7.07–7.04 (m, 1 H). ^{13}C-NMR (DMSO-*d*$_6$, 100 MHz): 189.26, 157.16, 155.30, 153.41, 140.87, 130.15, 130.01, 129.81, 129.34, 126.36, 125.82, 120.14, 115.09, 113.17. ESI-MS calculated for $C_{15}H_9Na_3O_{13}S_3$ [(M+Na)]$^+$, *m/z* 585.34, found [(M−3Na+3 HxA)+1HxA]$^+$, *m/z* 901.58.

(**6**). ^{1}H-NMR (DMSO-*d*$_6$, 400 MHz): 7.94–7.89 (m, 1 H), 7.61–7.50 (m, 3 H), 7.35 (d, 1 H, *J* = 2.24 Hz), 7.26–7.24 (m, 1 H), 7.15–7.13 (m, 1 H), 6.98 (d, 1 H, *J* = 8.32 Hz), 3.84 (s, 3 H), 3.81 (s, 3 H). ^{13}C-NMR (DMSO-*d*$_6$, 100 MHz): 188.90, 157.26, 153.63, 150.78, 149.08, 141.17, 130.02, 128.27, 126.225, 125.42, 123.28, 115.12, 113.17, 111.64, 110.33, 55.58. ESI-MS calculated for $C_{17}H_{14}Na_2O_{11}S_2$ [(M+Na)]$^+$, *m/z* 527.34, found [(M−2Na+2 HxA)+1HxA]$^+$, *m/z* 764.58.

(**7**). ^{1}H-NMR (DMSO-*d*$_6$, 400 MHz): 7.41 (d, 1 H, *J* = 8.36 Hz), 7.19 (d, 1 H, *J* = 2.84 Hz), 7.10 (d, 1 H, *J* = 16.00 Hz), 7.02–7.00 (m, 1 H), 6.90–6.85 (m, 2 H), 6.28 (d, 1 H, *J* = 2.16 Hz), 3.70 (s, 6 H), 3.62 (s, 3 H). ^{13}C-NMR (DMSO-*d*$_6$, 100 MHz): 192.22, 160.75, 157.68, 152.51, 150.33, 144.91, 143.42, 129.62, 127.78, 121.61, 120.19, 114.39, 111.66, 98.02, 93.43, 55.82, 55.70, 55.27. ESI-MS calculated for $C_{18}H_{16}Na_2O_{12}S_2$ [(M+Na)]$^+$, *m/z* 534.42, found [(M−2Na+2 HxA)+1HxA]$^+$, *m/z* 794.49.

(**8**). ^{1}H-NMR (DMSO-*d*$_6$, 400 MHz): 7.64 (s, 1 H), 7.29 (d, 1 H, *J* = 8.44 Hz), 7.10 (d, 1 H, *J* = 16.00 Hz), 7.00 (d, 1 H, *J* = 8.52 Hz), 6.92 (d, 1 H, *J* = 2.00 Hz), 6.67 (d, 1 H, *J* = 15.96 Hz), 6.36 (d, 1 H, *J* = 1.92 Hz), 3.79 (s, 3 H), 3.78 (s, 3 H), 3.70 (s, 3 H). ^{13}C-NMR (DMSO-*d*$_6$, 100 MHz): 192.57, 160.64, 157.47, 152.54, 152.18, 144.11, 142.83, 126.79, 126.68, 124.35, 120.25, 114.06, 112.66, 97.97, 93.27, 55.78, 55.74, 55.25. ESI-MS calculated for $C_{18}H_{16}Na_2O_{12}S_2$ [(M+Na)]$^+$, *m/z* 534.42, found [(M−2Na+2 HxA)+1HxA]$^+$, *m/z* 794.53.

(**9**). ^{1}H-NMR (DMSO-*d*$_6$, 400 MHz): 8.23–8.18 (m, 1 H), 7.76–7.72 (m, 1 H), 7.63–7.58 (m, 2 H), 7.51–7.49 (m, 2 H), 7.18 (m, 1 H), 7.09–7.06 (m, 2 H), 3.75 (s, 3 H). ^{13}C-NMR (DMSO-*d*$_6$, 100 MHz): 190.45, 153.84, 152.48, 142.34, 138.54, 132.17, 131.88, 131.02, 129.42, 126.56, 124.49, 123.18, 121.71, 118.26, 114.88, 56.09. ESI-MS calculated for $C_{16}H_{12}Na_2O_{10}S_2$ [(M+Na)]$^+$, *m/z* 474.37, found [(M−2Na+2 HxA)+1HxA]$^+$, *m/z* 734.46.

(**10**). ^{1}H-NMR (DMSO-d_6, 400 MHz): 7.88–7.82 (m, 3 H), 7.56 (d, 1 H, J = 8.64 Hz), 7.40 (m, 1 H), 7.32 (d, 1 H, J = 2.24 Hz), 7.15–7.08 (m, 2 H), 7.00 (t, 1 H, J = 7.56 Hz), 3.70 (s, 3 H). ^{13}C-NMR (DMSO-d_6, 100 MHz): 189.45, 158.01, 157.22, 153.43, 135.28, 131.53, 130.19, 130.02, 127.86, 127.17, 126.42, 123.69, 120.68, 115.06, 113.18, 111.61, 55.63. ESI-MS calculated for $C_{16}H_{12}Na_2O_{10}S_2$ $[(M+Na)]^+$, *m/z* 474.37, found $[(M-2Na+2\ HxA)+1HxA]^+$, *m/z* 734.46.

Sulfated flavonoid-quinazolinone hetero-dimers **(31–34) [3]**

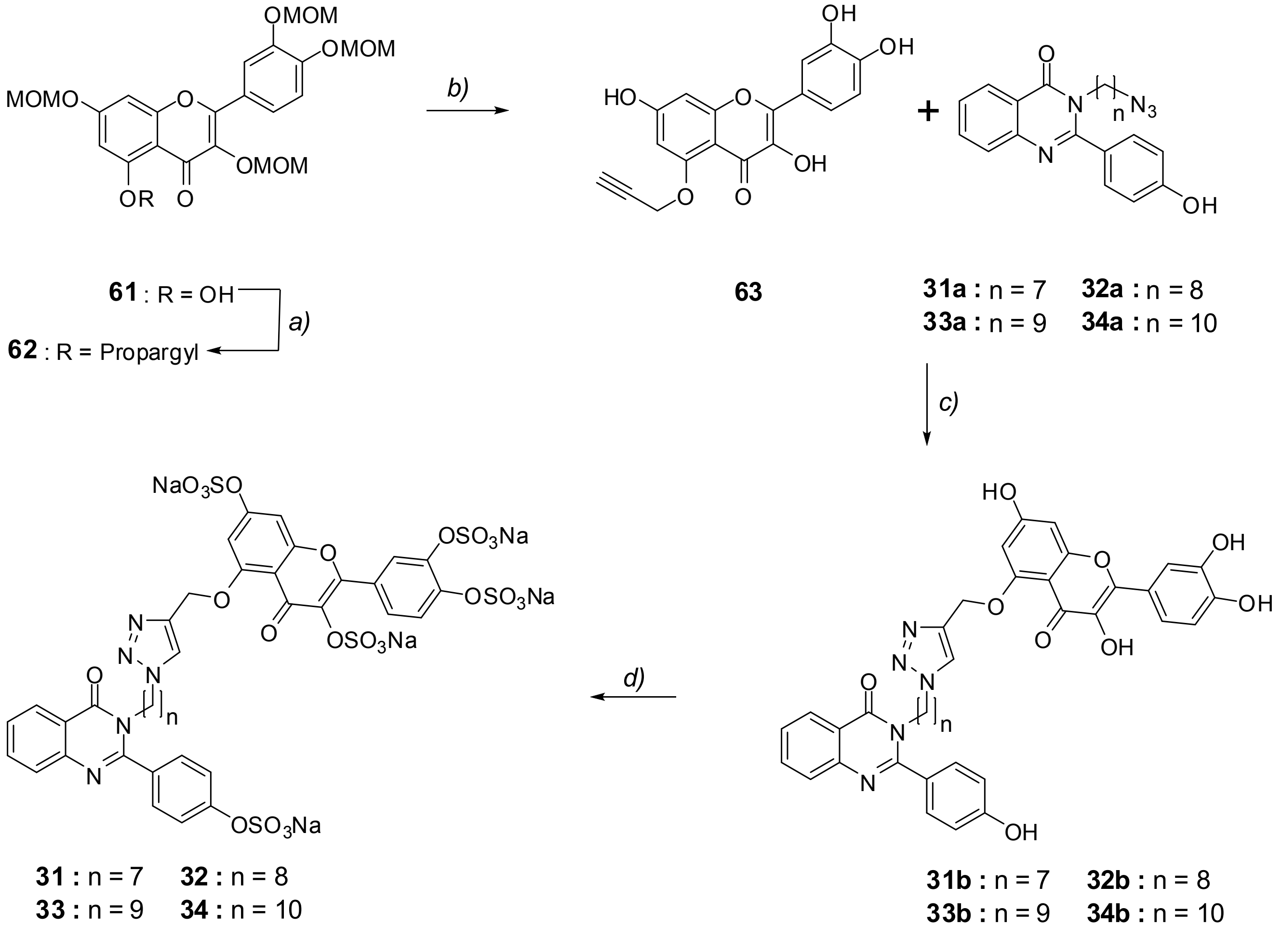

Scheme S2. *a)* K_2CO_3, propargyl bromide, DMF, rt/2 h, 85%–90%; *b)* 3N HCl, acetone, reflux/overnight, 55%–60%; *c)* $CuSO_4 \cdot 5H_2O$ (1 mol %), sodium ascorbate (5 mol %), DMF/H_2O (1:1), rt/overnight, 80%–95%; *d)* SO_3/Me_3N, Et_3N, CH_3CN, microwave/30 min, 85%–90%.

General procedure for synthesis of substituted phenyl quinazolin-4(3*H*)-one (58). To a stirred solution of anthranilamide (1.0 equiv) in anhydrous DMA, substituted benzaldehyde (1.1 equiv) and $NaHSO_3$ (1.5 equiv) was added in a single neck flask attached with a reflux condenser. The reaction mixture was vigorously stirred at 145 °C for 12 h; the reaction mixture was diluted with EtOAC (25 mL) and water (25 mL). The organic layer was separated and the aqueous phase was extracted with EtOAc (2 × 25 mL). The organic extracts were combined, washed with saturated NaCl solution (25 mL), and dried over anhydrous Na_2SO_4. Removal of the solvent under reduced pressure fallowed by the

purification of the crude by flash chromatography on silica gel (10%–80% ethyl acetate in hexanes) afforded 2-aryl quinazolin-4(3*H*)-one. Characterization data were reported earlier in reference 3.

General procedure for acetylation of hydroxyls in phenyl quinazolin-4(3*H*)-one core structure (59). To a solution of phenyl quinazolin-4(3*H*)-one in dry DCM was added pyridine (2.0 equiv per hydroxyl group) and acetic anhydride (1.0 equiv per hydroxyl group). After stirring for 2 h, the reaction mixture was diluted with EtOAC (25 mL) and water (25mL). The organic layer was separated and the aqueous phase was extracted with EtOAc (2 × 25 mL). The organic extracts were combined, washed with saturated 3N HCl (25mL) solution to remove excess pyridine and dried over anhydrous Na_2SO4. Removal of the solvent under reduced pressure afforded crude product and purified using flash chromatography on silica gel (10%–50% ethyl acetate in hexanes). Characterization data were reported earlier in reference 3.

General procedure for two steps synthesis of *N*^{3}-azide alkyl quinazolinon-4(3*H*)-one (31a–34a). To a solution of (1.0 equiv) in DMF was added K_2CO_3 (1.5 equiv) and stirred for two minutes. This was followed by addition of 1-bromo-n-chloroalkane (1.0 equiv) and stirred vigorously for 12 h. After the reaction completed as indicated from TLC the reaction mixture was diluted with EtOAC (25 mL) and water (25 mL). The organic layer was separated and the aqueous phase was extracted with EtOAc (2 × 25 mL) and removal of the solvent under reduced pressure afforded crude chloro-compounds which were directly used for next step without further purification. The chloro compound was then solubilized in DMF in a flask attached to a reflux condenser and sodium azide (1.5 equiv) was added to it. After stirring for overnight at 60 ºC, the reaction mixture was diluted with EtOAC (25 mL) and water. The organic layer was separated and the aqueous phase was extracted with EtOAc (2 × 25 mL). The organic extracts were combined, washed with saturated NaCl solution (25 mL), and dried over anhydrous Na_2SO_4. Removal of the solvent under reduced pressure afforded the desired crude azides which were further purified using flash chromatography on silica gel (20%–35% ethyl acetate in hexanes). Products used directly in the next reaction.

General procedure for protection of flavonoid by MOM (61). To a solution of flavonoid (1.0 equiv) in DCM, *N,N'*-diisopropylethylamine (8.0 equiv) and MOM chloride (3.5 equiv) was added under nitrogen. After vigorous stirring at 0 °C for 1 h, the reaction mixture was allowed to warm to room temperature over 2 h and the stirring was maintained for 12 h. The resulting mixture was diluted with water (100 mL), extracted with EtOAC (200 mL), and then the organic layer was washed with water (100 mL) and dried over Na_2SO_4. The residue obtained after removal of the solvent was purified by flash column chromatography. Characterization data were reported earlier in reference 3.

General procedure for flavonoid propargylation (62). To a solution of MOM-protected flavonoid in DMF was added K_2CO_3 (1.5 equiv) and allowed this reaction mixture to stir for 2 min fallowed by the addition of propargyl bromide (1.0 equiv). After stirring for 12 h at room temperature, the reaction mixture was diluted with EtOAC (25 mL) and water (25mL). The organic layer was separated and the aqueous phase was extracted with EtOAc (2 × 25 mL). The organic extracts were combined, washed with saturated NaCl solution (25 mL), and dried over anhydrous Na_2SO_4. Removal of the solvent under reduced pressure fallowed by purification using flash column chromatography afforded the desired propargylated compounds in quantitative yield.

(**62**). ^{1}H-NMR ($CDCl_3$, 400 MHz): 7.84 (d, J = 2.1 Hz, 1 H), 7.79–7.71 (m, 1 H), 7.29 (d, J = 8.8 Hz, 1 H), 6.63 (d, J = 2.1 Hz, 1 H), 6.47 (d, J = 2.1 Hz, 1 H), 5.32 (s, 2 H), 5.25 (s, 2H), 5.21 (s, 2 H), 5.20 (s, 2 H), 4.86 (d, J = 2.2 Hz, 2 H), 3.55 (s, 3 H), 3.51 (s, 3 H), 3.27 (s, 2 H), 3.25(s, 3 H), 2.53(t, J = 2.2 Hz, 1 H). MS (ESI) calculated for $C_{26}H_{28}O_{11}$ $[(M+H)]^+$, *m/z* 517.49, found *m/z* 539.45 $[(M+Na)]^+$.

General procedure of MOM deprotection (63). The compound was solubilized in acetone in a flask attached to a reflux condenser and 3N HCl was added to it. After stirring for 12 h at reflux temperature, the reaction mixture was neutralized with $NaHCO_3$ solution and diluted with EtOAC (25 mL). The organic layer was separated and the aqueous phase was extracted with EtOAc (2 × 25 mL). The organic extracts were combined, washed with saturated NaCl solution (25 mL), and dried over anhydrous Na_2SO_4. Removal of the solvent under reduced pressure afforded the desired crude which was further purified using flash chromatography.

(**63**). ^{1}H-NMR (DMSO-d_6, 400 MHz): 10.70 (s, 1 H), 9.37 (bs, 2 H), 8.58 (s, 1 H), 7.56 (d, J = 2.2 Hz, 1 H), 7.43–7.40 (m, 1 H), 6.80 (d, J = 8.5 Hz, 1 H), 6.45–6.38 (m, 2 H), 4.80 (d, J = 2.2 Hz, 2 H), 3.56 (t, J = 2.2 Hz, 1 H). ^{13}C-NMR (100 MHz, DMSO-d_6): 170.77, 162.06, 158.09, 157.82, 146.98, 145.03, 142.05, 137.06, 122.19, 119.16, 115.58, 114.51, 105.53, 98.17, 95.52, 78.77, 78.68, 56.53. MS (ESI) calculated for $C_{18}H_{12}O_7$ $[(M+H)]^+$, *m/z* 341.28, found *m/z* 363.22 $[(M+Na)]^+$.

General procedure for copper-catalyzed azide alkyne cycloaddition (1,4-cycloaddition) (31b–34b). To a solution of terminal alkyne (1 equiv.) and azide (1.0 equiv) were suspended in 1:1 mixture of H_2O and DMF. Freshly prepared sodium ascorbate solution in water (5 mol %) was added fallowed by $CuSO_4.5H_2O$ solution in water (1 mol %) was added. The heterogeneous reaction mixture was stirred vigorously for 12 h, at which point it cleared and TLC analysis indicated complete consumption of the reactants. To this reaction mixture, 2 mL of 3% ammonia solution was added for quenching of excess $CuSO_4.5H_2O$ and stirred for further 10 min. The reaction mixture was diluted with EtOAC (25 mL), stirred for another 10–15 min and then filtered through a Celite bed. The combined reaction mixture was extracted with EtOAc (2 × 25 mL) and removal of the solvent under reduced pressure afforded crude compound which was further purified using flash chromatography.

(**31b**). ^{1}H-NMR (DMSO-d_6, 400 MHz): 10.67 (s, 1 H), 9.89 (s, 1 H), 9.37 (s, 1 H), 9.14 (s, 1 H), 8.51 (s, 1 H), 8.3 (t, J = 3.6 Hz, 2 H,), 8.18 (s, 1 H), 8.13 (d, J = 3.3 Hz, 1 H), 7.82–7.80 (m, 2 H), 7.5–7.4 (m, 3 H), 6.85–6.78 (m, 3 H), 6.42 (d, J = 1.7 Hz, 2 H), 5.15 (s, 2 H), 4.60 (t, J = 3.7 Hz, 2 H), 4.30 (t, J = 3.7 Hz, 2 H), 1.81–1.76 (m, 4 H), 1.19–1.09 (m, 6 H). ^{13}C-NMR (100 MHz, DMSO-d_6): 170.84, 166.05, 162.29, 160.06, 159.17, 158.99, 157.86, 151.34, 146.96, 145.03, 142.69, 141.96, 137.05, 133.94, 129.86, 128.41, 127.25, 126.28, 124.10, 123.13, 122.22, 119.14, 115.57, 115.29, 114.49, 114.21, 97.81, 95.16, 66.54, 52.80, 49.39, 35.74, 29.69, 28.54, 28.26, 28.13, 25.76, 25.44. MS (ESI) calculated for $C_{39}H_{35}N_5O_9$ $[(M+H)]^+$, *m/z* 718.72, found *m/z* 740.67 $[(M+Na)]^+$.

(**32b**). ^{1}H-NMR (DMSO-d_6, 400 MHz): 10.65 (s, 1 H), 9.85 (s, 1 H), 9.34 (s, 1 H), 9.12 (s, 1 H), 8.50 (s, 1 H), 8.23 (t, J = 3.6 Hz, 2 H,), 8.14 (s, 1 H), 8.09 (d, J = 3.3 Hz, 1 H), 7.81–7.78 (m, 2 H), 7.5–7.4 (m, 3 H), 6.84–6.77 (m, 3 H), 6.41 (d, J = 1.7 Hz, 2 H), 5.15 (s, 2 H), 4.59 (t, J = 3.6 Hz, 2 H), 4.28 (t, J = 3.6 Hz, 2 H), 1.84–1.79 (m, 4 H), 1.18–1.09 (m, 8 H). ^{13}C-NMR (100 MHz, DMSO-d_6): 170.89, 166.08, 162.28, 160.06, 159.16, 158.98, 157.87, 151.34, 146.98, 145.08, 142.69, 141.98, 137.08, 133.98,

129.89, 128.41, 127.28, 125.98, 124.12, 123.18, 122.80, 119.18, 115.58, 115.35, 114.49, 114.26, 97.89, 95.18, 66.54, 52.80, 49.38, 35.80, 29.69, 28.84, 28.27, 28.18, 25.77, 25.48. MS (ESI) calculated for $C_{40}H_{37}N_5O_9$ $[(M+H)]^+$, *m/z* 732.75, found *m/z* 754.69 $[(M+Na)]^+$.

(**33b**). ^{1}H-NMR (DMSO-d_6, 400 MHz): 10.61 (s, 1 H), 9.84 (s, 1 H), 9.31 (s, 1 H), 9.10 (s, 1 H), 8.50 (s, 1 H), 8.30 (t, *J* = 3.7 Hz, 2 H,), 8.13 (s, 1 H), 8.01 (d, *J* = 3.2 Hz, 1 H), 7.81–7.79 (m, 2 H), 7.56–7.50 (m, 3 H), 6.84–6.78 (m, 3 H), 6.42 (d, *J* = 1.7 Hz, 2 H), 5.15 (s, 2 H), 4.60 (t, *J* = 3.7 Hz, 2 H), 4.29 (t, *J* = 3.7 Hz, 2 H), 1.84–1.79 (m, 4 H), 1.22–1.10 (m, 10 H). ^{13}C-NMR (100 MHz, DMSO-d_6): 170.86, 166.09, 162.29, 159.96, 159.18, 158.97, 157.80, 151.38, 146.97, 145.12, 143.13, 141.89, 137.09, 133.97, 129.59, 128.81, 127.29, 125.92, 124.15, 123.98, 122.78, 119.89, 114.98, 115.65, 114.39, 114.86, 97.89, 95.28, 66.64, 52.85, 49.28, 35.79, 28.89, 28.72, 28.28, 28.13, 25.93, 25.46, 25.41. MS (ESI) calculated for $C_{41}H_{39}N_5O_9$ $[(M+H)]^+$, *m/z* 746.78, found *m/z* 768.72 $[(M+Na)]^+$.

(**34b**). ^{1}H-NMR (DMSO-d_6, 400 MHz): 10.63 (s, 1 H), 9.86 (s, 1 H), 9.34 (s, 1 H), 9.12 (s, 1 H), 8.53 (s, 1 H), 8.30 (d, *J* = 3.7 Hz, 2 H,), 8.13 (s, 1 H), 8.02 (d, *J* = 3.2 Hz, 1 H), 7.81–7.79 (m, 2 H), 7.56–7.49 (m, 3 H), 6.84–6.78 (m, 3 H), 6.46–6.41 (m, 2 H), 5.15 (s, 2 H), 4.60 (t, *J* = 3.6 Hz, 2 H), 4.29 (t, *J* = 3.6 Hz, 2 H), 1.81–1.75 (m, 4 H), 1.22–1.10 (m, 12 H). ^{13}C-NMR (100 MHz, DMSO-d_6): 171.06, 166.19, 163.01, 158.08, 159.12, 158.97, 157.76, 151.38, 146.89, 145.32, 143.33, 142.03, 137.09, 133.98, 129.61, 129.31, 127.89, 125.39, 123.91, 123.98, 123.68, 119.49, 114.68, 115.85, 114.89, 114.88, 97.39, 94.88, 66.34, 52.86, 49.38, 35.98, 28.01, 28.78, 28.19, 28.14, 25.98, 25.48, 25.43. MS (ESI) calculated for $C_{42}H_{41}N_5O_9$ $[(M+H)]^+$, *m/z* 760.80, found *m/z* 782.76 $[M+Na]^+$.

General procedure for chemical sulfation of flavonoid-quinazolinoe *hetero*-dimers (31–34). See above. Characterization data were reported earlier in reference 3 for (**28**–**30**)

(**31**). ^{1}H-NMR (DMSO-d_6, 400 MHz): 8.42 (d, *J* = 8.7 Hz, 2 H), 8.31–8.28 (m, 1 H), 8.15–8.11 (m, 3 H), 7.93–7.9 (m, 2 H), 7.65–7.62 (m, 2 H), 7.34 (d, *J* = 8.7 Hz, 2 H), 7.13–7.07 (m, 1 H), 6.85–6.81 (m, 1 H), 5.24 (s, 2 H), 4.71 (t, *J* = 6.4 Hz, 2 H), 4.37 (t, *J* = 7.9 Hz, 2 H), 1.93–1.85 (m, 4 H) 1.55–1.24 (m, 6 H). ^{13}C-NMR (100 MHz, D_2O): 169.04, 160.39, 157.96, 155.86, 154.12, 148.67, 143.76, 135.18, 132.09, 129.78, 128.80, 127.34, 124.34, 123.16, 119.08, 115.13, 114.19, 114.03, 113.96, 66.76, 52.80, 49.42, 29.23, 28.61, 28.35, 25.29, 25.18. MS (ESI) calculated for $C_{39}H_{30}N_5Na_5O_{24}S_5$ $[M-Na]^-$, *m/z* 1203.94, found $[M-2Na]^{2-}$, *m/z* 590.38.

(**32**). ^{1}H-NMR (DMSO-d_6, 400 MHz): 8.44 (d, *J* = 8.8 Hz, 2 H), 8.32–8.27 (m, 1 H), 8.15–8.12 (m, 3 H), 7.94–7.92 (m, 2 H), 7.64–7.61 (m, 2 H), 7.34 (d, *J* = 8.8 Hz, 2 H), 7.13–7.07 (m, 1 H), 6.86–6.82 (m, 1 H), 5.23 (s, 2 H), 4.71 (t, *J* = 6.4 Hz, 2 H), 4.38 (t, *J* = 7.9 Hz, 2 H), 1.93–1.85 (m, 4 H) 1.54–1.23 (m, 8 H). ^{13}C-NMR (100 MHz, D_2O): 169.03, 160.23, 158.86, 156.07, 151.06, 148.58, 142.20, 134.15, 131.82, 129.96, 128.96, 127.30, 126.79, 124.30, 123.21, 119.83, 115.34, 114.37, 114.17, 66.81, 52.81, 49.40, 29.70, 28.54, 28.27, 28.12, 25.78, 25.44. MS (ESI) calculated for $C_{40}H_{32}N_5Na_5O_{24}S_5$ $[(M-Na)]^-$, *m/z* 1217.95, found $[(M-2Na)]^{2-}$, *m/z* 597.39.

(**33**). ^{1}H-NMR (DMSO-d_6, 400 MHz): 8.45 (d, *J* = 8.7 Hz, 2 H), 8.30–8.27 (m, 1 H), 8.16–8.13 (m, 3 H), 7.94–7.92 (m, 2 H), 7.63–7.61 (m, 2 H), 7.34 (d, *J* = 8.8 Hz, 2 H), 7.13–7.07 (m, 1 H), 6.84 (d, *J* = 2.1 Hz, 1 H), 5.23 (s, 2 H), 4.71 (t, *J* = 6.4 Hz, 2 H), 4.37 (t, *J* = 7.3 Hz, 2 H), 1.93–1.84 (m, 4 H)

1.54–1.28 (m, 10 H). ^{13}C-NMR (100 MHz, D_2O): 169.24, 158.86, 156.10, 155.06, 154.18, 153.33, 150.18, 148.58, 143.76, 134.11, 134.38, 129.94, 128.95, 127.33, 124.29, 123.21, 119.81, 115.33, 114.38, 66.04, 52.78, 49.39, 29.71, 28.74, 28.29, 28.14, 25.82, 25.46, 25.18, 24.39, 24.85. MS (ESI) calculated for $C_{41}H_{34}N_5Na_2O_{24}S_5$ $[(M{-}Na)]^-$, *m/z* 1231.97, found $[(M{-}Na)]^-$, *m/z* 604.38.

(**34**). ^{1}H-NMR (DMSO-d_6, 400 MHz): 8.44 (d, *J* = 8.6 Hz, 2 H), 8.30–8.27 (m, 1 H), 8.17–8.13 (m, 3 H), 7.93–7.91 (m, 2 H), 7.62–7.6 (m, 2 H), 7.32 (d, *J* = 8.8 Hz, 2 H), 7.13–7.07 (m, 1 H), 6.85 (d, *J* = 2.2 Hz, 1 H), 5.23 (s, 2 H), 4.72 (t, *J* = 6.3 Hz, 2 H), 4.38 (t, *J* = 7.4 Hz, 2 H), 1.94–1.84 (m, 4 H) 1.54–1.28 (m, 12 H). ^{13}C-NMR (100 MHz, D_2O): 169.28, 160.13, 158.26, 156.07, 151.08, 148.53, 142.20, 134.18, 131.72, 129.86, 128.64, 127.34, 126.76, 124.08, 123.23, 119.82, 115.28, 114.38, 114.13, 66.78, 52.13, 49.31, 29.73, 28.28, 28.37, 28.24, 25.14, 25.48, 24.19**.** MS (ESI) calculated for $C_{42}H_{36}N_5Na_2O_{24}S_5$ $[(M{-}Na)]^-$, *m/z* 1245.98, found $[(M{-}2Na)]^{2-}$, *m/z* 612.43.

Sulfated quinazolinone homo-dimers (42–47) [3]

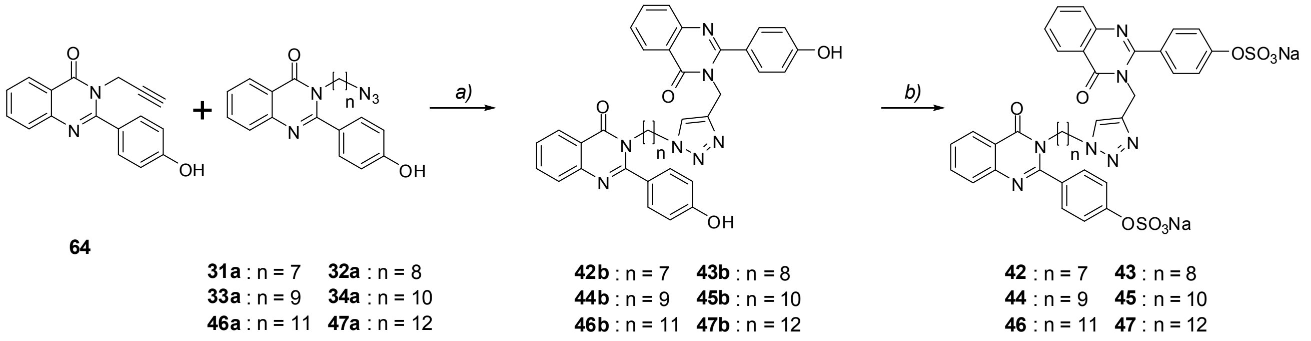

Scheme S3. *a)* $CuSO_4{\cdot}5H_2O$ (1 mol %), sodium ascorbate (5 mol %), DMF/H_2O (1:1), rt, overnight, 80%–95%; *b)* SO_3:Me_3N, TEA, CH_3CN, microwave, 30 min–2 h, 90 °C, 85%–90%.

General procedure for synthesis of substituted phenyl quinazolin-4(3*H*)-one. See above.

General procedure for protection of hydroxyls in phenyl quinazolin-4(3*H*)-one core structure. See above.

General procedure for synthesis of the propargylated quinazolinone monomer (64). To a solution of quinazolinone monomer in DMF was added K_2CO_3 (1.5 equiv) and allowed this reaction mixture to stir for 2 min fallowed by the addition of propargybromide (1.5 equiv). After stirring for 3 h, the reaction mixture was diluted with EtOAC (25 mL) and water (25 mL). The organic layer was separated and the aqueous phase was extracted with EtOAc (2 × 25 mL). The organic extracts were combined, washed with saturated NaCl solution (25 mL), and dried over anhydrous Na_2SO_4. Removal of the solvent under reduced pressure afforded the desired propargylated compounds in quantitative yield and sufficient purity (as indicated by TLC) to be directly used in the next reaction without any further purification. The crude recation mixture was then subjected to deacetylation by solubilizing in THF followed by addition of lithium hydroxide monohydrate Li(OH).H_2O (2 equiv). After stirring for overnight, the reaction mixture was diluted with EtOAC (25 mL) and water (25mL). The organic layer was separated and the aqueous phase was extracted with EtOAc (2 × 25 mL) and removal of the solvent under reduced pressure afforded crude deacetylated compounds which were further purified using flash

chromatography on silica gel (20%–35% ethyl acetate in hexanes). Characterization data were reported earlier in reference 3.

General procedure for two steps synthesis of *N*^{3}-azide alkyl quinazolinon-4(3*H*)-one (31a–34a) and 46a and 47a. See above. Compounds used as such in next reaction.

General procedure for copper-catalyzed azide alkyne cycloaddition (1,4-cycloaddition) (42b–47b). See above.

(**42b**). ^{1}H-NMR ($(CD_3)_2CO$, 400 MHz): 8.65 (s, 1 H), 8.43–8.35 (m, 4 H), 8.04–7.96 (m, 3 H), 7.72–7.75 (m, 4 H), 7.4–7.37 (m, 2 H), 6.87–6.82 (m, 4 H), 5.74 (s, 2 H), 4.56 (t, *J* = 3.5 Hz, 2 H), 4.30 (t, *J* = 3.5 Hz, 2 H), 1.83–1.75 (m, 4 H), 1.40–1.23 (m, 6 H). ^{13}C-NMR ($(CD_3)_2CO$, 100MHz): 167.41, 166.87, 160.97, 160.87, 160.58, 160.40, 153.08, 152.95, 143.66, 134.59, 134.39, 131.15, 131.04, 130.66, 128.48, 126.94, 126.83, 124.88, 124.18, 116.11, 116.02, 115.93, 115.73, 115.59, 67.53, 60.99, 50.63, 30.88, 27.05, 26.61. MS (ESI) calculated for $C_{38}H_{35}N_7O_4$ $[(M+H)]^+$, m/*z* 654.73, found *m/z* 676.71 $[(M+Na)]^+$.

(**43b**). ^{1}H-NMR ($(CD_3)_2CO$, 400MHz): 8.43–8.34 (m, 4 H), 8.05–7.96 (m, 3 H), 7.83–7.71 (m, 5 H), 7.41–7.38 (m, 2 H), 6.87–6.82 (m, 4 H), 5.75 (s, 2 H), 4.59 (t, *J* = 3.6 Hz, 2 H), 4.29 (t, *J* = 3.6 Hz, 2 H), 1.83–1.77 (m, 4 H), 1.43–1.21 (m, 8 H). ^{13}C-NMR ($(CD_3)_2CO$, 100MHz): 166.86, 160.82, 160.38, 153.09, 152.98, 143.78, 134.61, 134.42, 131.14, 131.03, 130.61, 130.45, 128.48, 126.95, 126.84, 124.91, 124.18, 116.05, 115.96, 115.73, 115.59, 67.56, 60.97, 50.65, 29.98, 29.79, 27.06, 26.66. MS (ESI) calculated for $C_{39}H_{37}N_7O_4$ $[(M+H)]^+$, m/*z* 668.29, found *m/z* 690.22 $[(M+Na)]^+$.

(**44b**). ^{1}H-NMR ($(CD_3)_2CO$, 400MHz): 8.77 (s, 1 H), 8.41–8.34 (m, 4 H), 8.03–7.94 (m, 3 H), 7.73–7.69 (m, 4 H), 7.41–7.36 (m, 2 H), 6.86–6.82 (m, 4 H), 5.73 (s, 2 H), 4.56 (t, *J* = 3.7 Hz, 2 H), 4.26 (t, *J* = 3.7 Hz, 2 H), 1.80–1.71 (m, 4 H), 1.42–1.15 (m, 10 H). ^{13}C-NMR ($(CD_3)_2CO$, 100MHz): 167.40, 166.84, 162.84, 160.99, 160.90, 160.58, 160.38, 153.05, 152.93, 143.66, 134.61, 134.42, 131.15, 131.05, 130.60, 130.41, 130.20, 128.47, 126.95, 126.83, 124.95, 124.19, 116.40, 116.11, 116.03, 115.72, 115.56, 67.61, 60.94, 50.68, 36.18, 30.21, 27.05, 26.74. MS (ESI) calculated for $C_{40}H_{39}N_7O_4$ $[(M+H)]^+$, m/*z* 682.78, found *m/z* 704.73 $[(M+Na)]^+$.

(**45b**). ^{1}H-NMR ($(CD_3)_2CO$, 400 MHz): 8.71 (s, 1 H), 8.42–8.34 (m, 4 H), 8.04–7.96 (m, 3 H), 7.74–7.71 (m, 4 H), 7.40–7.36 (m, 2 H), 6.86–6.82 (m, 4 H), 5.75 (s, 2 H), 4.59 (t, *J* = 3.5 Hz, 2 H), 4.27 (t, *J* = 3.5 Hz, 2 H), 1.83–1.72 (m, 4 H), 1.43–1.14 (m, 12 H). ^{13}C-NMR ($(CD_3)_2CO$, 100 MHz): 167.42, 166.86, 162.74, 160.89, 160.58, 160.38, 160.38, 153.06, 152.95, 143.64, 134.62, 134.42, 131.15, 131.04, 130.61, 130.44, 128.478, 126.95, 126.83, 124.93, 124.19, 116.08, 116.00, 115.73, 115.57, 67.59, 60.95, 50.66, 36.12, 27.08, 26.74. MS (ESI) calculated for $C_{41}H_{41}N_7O_4$ $[(M+H)]^+$, m/*z* 696.81, found *m/z* 718.79 $[(M+Na)]^+$.

(**46b**). ^{1}H-NMR ($(CD_3)_2CO$, 400 MHz): 8.71 (s, 1 H), 8.41–8.33 (m, 4 H), 8.05–7.94 (m, 3 H), 7.74–7.71 (m, 4 H), 7.40–7.36 (m, 2 H), 6.87–6.83 (m, 4 H), 5.3 (s, 2 H), 4.58 (t, *J* = 3.6 Hz, 2 H), 4.26 (t, *J* = 3.6 Hz, 2 H), 1.81–1.74 (m, 4 H), 1.42–1.13 (m, 14 H). ^{13}C-NMR ($(CD_3)_2CO$, 100 MHz): 167.41, 166.58, 162.77, 160.89, 160.58, 160.38, 160.29, 153.08, 152.97, 143.69, 135.02, 134.48, 132.18, 131.04, 130.68, 130.24, 128.49, 126.98, 126.85, 124.98, 124.08, 116.09, 116.00, 115.78, 115.37, 67.89, 60.98, 50.68, 36.13, 27.09, 26.78. MS (ESI) calculated for $C_{42}H_{43}N_7O_4$ $[(M+H)]^+$, m/*z* 710.84, found *m/z* 732.81 $[(M+Na)]^+$.

(**47b**). ^{1}H-NMR ($(CD_3)_2CO$, 400 MHz): 8.72 (s, 1 H), 8.41–8.33 (m, 4 H), 8.4–7.96 (m, 3 H), 7.75–7.70 (m, 4 H), 7.42–7.34 (m, 2 H), 6.86–6.82 (m, 4 H), 5.3 (s, 2 H), 4.58 (t, J = 3.6 Hz, 2 H), 4.26 (t, J = 3.6 Hz, 2 H), 1.82–1.74 (m, 4 H), 1.42–1.12 (m, 16 H). ^{13}C-NMR ($(CD_3)_2CO$, 100 MHz): 167.42, 166.47, 162.78, 160.85, 160.54, 160.32, 160.24, 153.28, 152.77, 143.09, 135.08, 134.65, 132.18, 131.24, 130.68, 130.41, 128.49, 126.09, 126.84, 124.96, 124.18, 116.21, 116.05, 115.72, 114.92, 67.85, 60.92, 50.65, 36.14, 27.08, 26.77. MS (ESI) calculated for $C_{43}H_{45}N_7O_4$ [(M+H)]$^+$, m/z 724.86, found *m/z* 746.82 [(M+Na)]$^+$.

General procedure for copper-catalyzed azide alkyne cycloaddition (1,5-cycloaddition). Involved in synthesis of sulfated molecule (40). A solution of a terminal azide (1 equiv) and a terminal alkyne (1 equiv) in 0.5 mL of dioxane was added to Cp*RuCl(PPh3)2 (2 mol%) dissolved in 2.5 mL of dioxane. The vial was purged with nitrogen, sealed, and heated in an oil bath at 60 °C for 12 h, at which TLC indicated complete consumption of the alkyne and the azide starting materials. The mixture was adsorbed on to silica and chromatographed with hexanes/ethyl acetate to elute the product quantitative yield. Characterization data were reported earlier in reference 3.

General procedure for synthesis of *bis*-azide derivative. Involved in synthesis of sulfated molecule (41). To a solution of alkyne (2.0 equiv) and bis-azide (1.0 equiv) were suspended in 1:1 mixture of H_2O and DMF. Freshly prepared sodium ascorbate solution in water (10 mol %) was added fallowed by $CuSO_4.5H_2O$ solution in water (2 mol %) was added. The heterogeneous reaction mixture was stirred vigorously for 12 h, at which point it cleared and TLC analysis indicated complete consumption of the reactants. To this reaction mixture, 2 mL of 3% ammonia solution was added for quenching of excess $CuSO_4.5H_2O$ and stirred for further10 min. The reaction mixture was diluted with EtOAC (25 mL), stirred for another 10-15 min and then filtered through a Celite bed. The combined reaction mixture was extracted with EtOAc (2 × 25 mL) and removal of the solvent under reduced pressure afforded crude compound which was further purified using flash chromatography. Characterization data were reported earlier in reference 3.

General procedure for chemical sulfation of bis-quinazolinone *homo*-dimers (42–47). See above. Characterization data were reported earlier in reference 3 for (**35–39**).

(**42**). ^{1}H-NMR (D_2O, 400 MHz): 7.93 (d, J = 8.6 Hz, 2 H), 7.67–7.73 (m, 3 H), 7.25–7.12 (m, 9 H), 6.95 (d, J = 7.9 Hz, 1 H), 6.75–6.69 (m, 2 H), 5.19 (s, 2 H), 4.15 (bs, 2 H), 3.58 (bs, 2 H), 1.47 (s, 2 H), 0.98 (s, 2 H), 0.71–0.67 (m, 6 H). ^{13}C-NMR (100 MHz, D_2O): 165.70, 165.23, 158.71, 158.55, 153.69, 153.64, 150.06, 149.72, 142.81, 133.97, 133.77, 129.76, 129.63, 126.49, 125.80, 124.48, 121.10, 120.91, 113.64, 113.59, 66.72, 59.69, 50.31, 38.69, 29.38, 28.02, 27.64, 25.68, 25.16. MS (ESI) calculated for $C_{38}H_{33}N_7Na_2O_{10}S_2$ [(M−Na)]$^-$, *m/z* 834.16, found [(M−2Na)]$^{2-}$, *m/z* 405.85.

(**43**). ^{1}H-NMR (D_2O, 400 MHz): 7.94 (d, J = 8.5 Hz, 2 H), 7.78–7.73 (m, 3 H), 7.25–7.01 (m, 9 H), 7.02 (d, J = 7.9 Hz, 1 H), 6.78–6.74 (m, 2 H), 5.22 (s, 2 H), 4.12 (bs, 2 H), 3.62 (bs, 2 H), 1.44 (bs, 2 H), 1.02 (s, 2 H), 0.71–0.60 (m, 8 H). ^{13}C-NMR (100 MHz, D_2O): 165.85, 165.42, 158.72, 153.83, 153.67, 153.43, 150.12, 149.53, 142.92, 134.12, 133.67, 129.86, 129.72, 126.12, 125.58, 122.69, 121.20, 120.99, 113.79, 113.37, 66.78, 59.86, 50.35, 38.63, 29.49, 28.55, 28.28, 27.94, 25.80, 25.30. MS (ESI) calculated for $C_{39}H_{35}N_7Na_2O_{10}S_2$ [(M−Na)]$^-$, *m/z* 848.18, found [(M−2Na)]$^{2-}$, *m/z* 412.47.

(**44**). ^{1}H-NMR (D_2O, 400 MHz): 7.96 (d, J = 8.4 Hz, 2 H), 7.82–7.75 (m, 3 H), 7.25–7.19 (m, 9 H), 7.07 (d, J = 7.7 Hz, 1 H), 6.80–6.79 (m, 2 H), 5.25 (s, 2 H), 4.19 (bs, 2 H), 3.70 (bs, 2 H), 1.44 (bs, 2 H), 1.1 (s, 2 H), 0.70–0.56 (m, 10 H). ^{13}C-NMR (100 MHz, D_2O): 165.78, 165.43, 158.69, 154.03, 153.89, 153.83, 151.02, 149.84, 143.09, 134.85, 133.87, 129.69, 129.38, 126.82, 125.76, 123.79, 121.80, 120.99, 113.60, 113.34, 66.95, 60.09, 50.89, 38.68, 30.01, 28.58, 28.34, 27.86, 26.34, 25.52, 25.12. MS (ESI) calculated for $C_{40}H_{37}N_7Na_2O_{10}S_2$ $[(M-Na)]^{-}$, m/z 862.19, found $[(M-2Na)]^{2-}$, m/z 419.96.

(**45**). ^{1}H-NMR (D_2O, 400 MHz): 7.97 (d, J = 7.3 Hz, 2 H), 7.86–7.78 (m, 3 H), 7.29–7.19 (m, 9 H), 7.12 (d, J = 7.6 Hz, 1 H), 6.83–6.80 (m, 2 H), 5.28 (s, 2 H), 4.12 (bs, 2 H), 3.73 (bs, 2 H), 1.45 (bs, 2 H), 1.12 (s, 2 H), 0.74–0.53 (m, 12 H). ^{13}C-NMR (100 MHz, D_2O): 165.84, 165.43, 158.62, 154.09, 153.93, 153.09, 150.17, 149.08, 142.78, 134.89, 133.90, 129.83, 129.37, 126.08, 125.04, 123.79, 121.17, 120.96, 113.78, 113.34, 66.89, 60.18, 50.26, 42.53, 29.63, 28.93, 28.54, 25.96, 25.56, 25.12, 24.79. MS (ESI) calculated for $C_{41}H_{39}N_7Na_2O_{10}S_2$ $[(M-Na)]^{-}$, m/z 876.21, found $[(M-2Na)]^{2-}$, m/z 426.94.

(**46**). ^{1}H-NMR (D_2O, 400 MHz): 7.98 (d, J = 7.9 Hz, 2 H), 7.85–7.77 (m, 3 H), 7.31–7.23 (m, 9 H), 7.15 (d, J = 7.8 Hz, 1 H), 6.78–6.72 (m, 2 H), 5.39 (s, 2 H), 4.18 (bs, 2 H), 3.81 (bs, 2 H), 1.48 (bs, 2 H), 1.14 (s, 2 H), 0.89–0.68 (m, 14 H). ^{13}C-NMR (100 MHz, D_2O): 165.78, 165.39, 158.76, 155.06, 154.12, 153.67, 150.17, 149.01, 143.76, 135.18, 134.09, 129.78, 129.1, 126.64, 125.34, 123.76, 121.08, 120.13, 113.89, 113.18, 66.76, 60.04, 50.28, 42.89, 29.83, 28.68, 28.35, 25.29, 25.18, 25.12, 24.89, 24.65. MS (ESI) calculated for $C_{42}H_{41}N_7Na_2O_{10}S_2$ $[(M-Na)]^{-}$, m/z 890.23, found $[(M-2Na)]^{2-}$, m/z 433.53.

(**47**). ^{1}H-NMR (D_2O, 400 MHz): 7.96 (d, J = 8.3 Hz, 2 H), 7.89–7.81 (m, 3 H), 7.43–7.19 (m, 9 H), 7.03 (d, J = 7.8 Hz, 1 H), 6.83–6.75 (m, 2 H), 5.25 (s, 2 H), 4.13 (bs, 2 H), 3.84 (bs, 2 H), 1.48 (bs, 2 H), 1.25 (s, 2 H), 0.88–0.65 (m, 16 H). ^{13}C-NMR (100 MHz, D_2O): 165.78, 165.39, 158.76, 155.06, 154.12, 153.67, 150.17, 149.01, 143.76, 135.18, 134.09, 129.78, 129.1, 126.64, 125.34, 123.76, 121.08, 120.13, 113.89, 113.18, 66.76, 60.04, 50.28, 42.89, 29.83, 28.68, 28.35, 25.29, 25.18, 25.12, 24.89, 24.65. MS (ESI) calculated for $C_{43}H_{43}N_7Na_2O_{10}S_2$ $[(M-Na)]^{-}$, m/z 904.24, found $[(M-2Na)]^{2-}$, m/z 440.55.

Sulfated flavonoid homo-dimers **(48–55)**

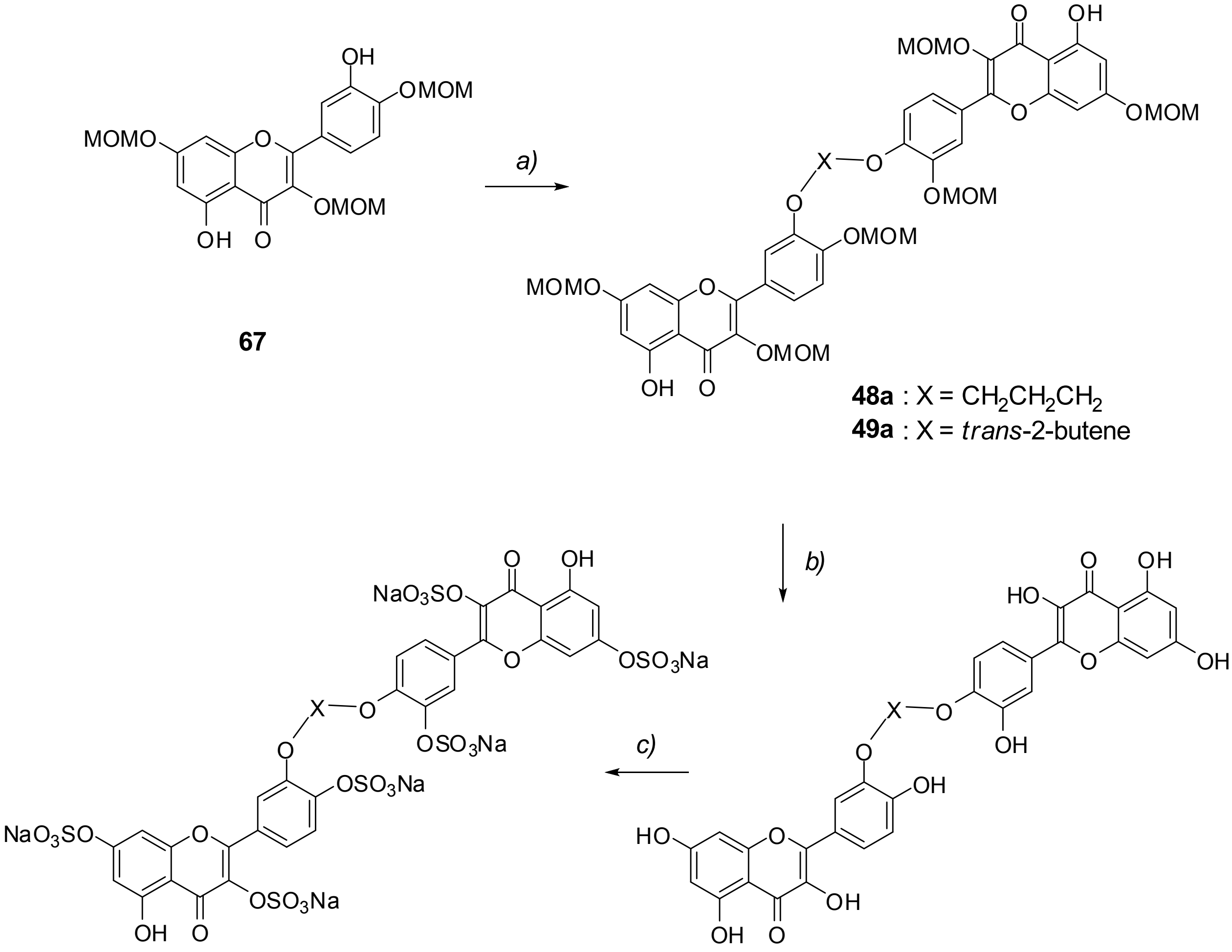

67

48a : X = $CH_2CH_2CH_2$
49a : X = *trans*-2-butene

48 : X = $CH_2CH_2CH_2$
49 : X = *trans*-2-butene

48b : X = $CH_2CH_2CH_2$
49b : X = *trans*-2-butene

Scheme S4. *a)* K_2CO_3, dibromoalkane (0.5 equiv), DMF, rt/3 h, 85%–90%, *b)* *p*-Toluenesulfonic acid, MeOH, reflux/48 h, 55%–65%, *c)* SO_3:Me_3N, Et_3N, CH_3CN, microwave/6 h, 75%–85%.

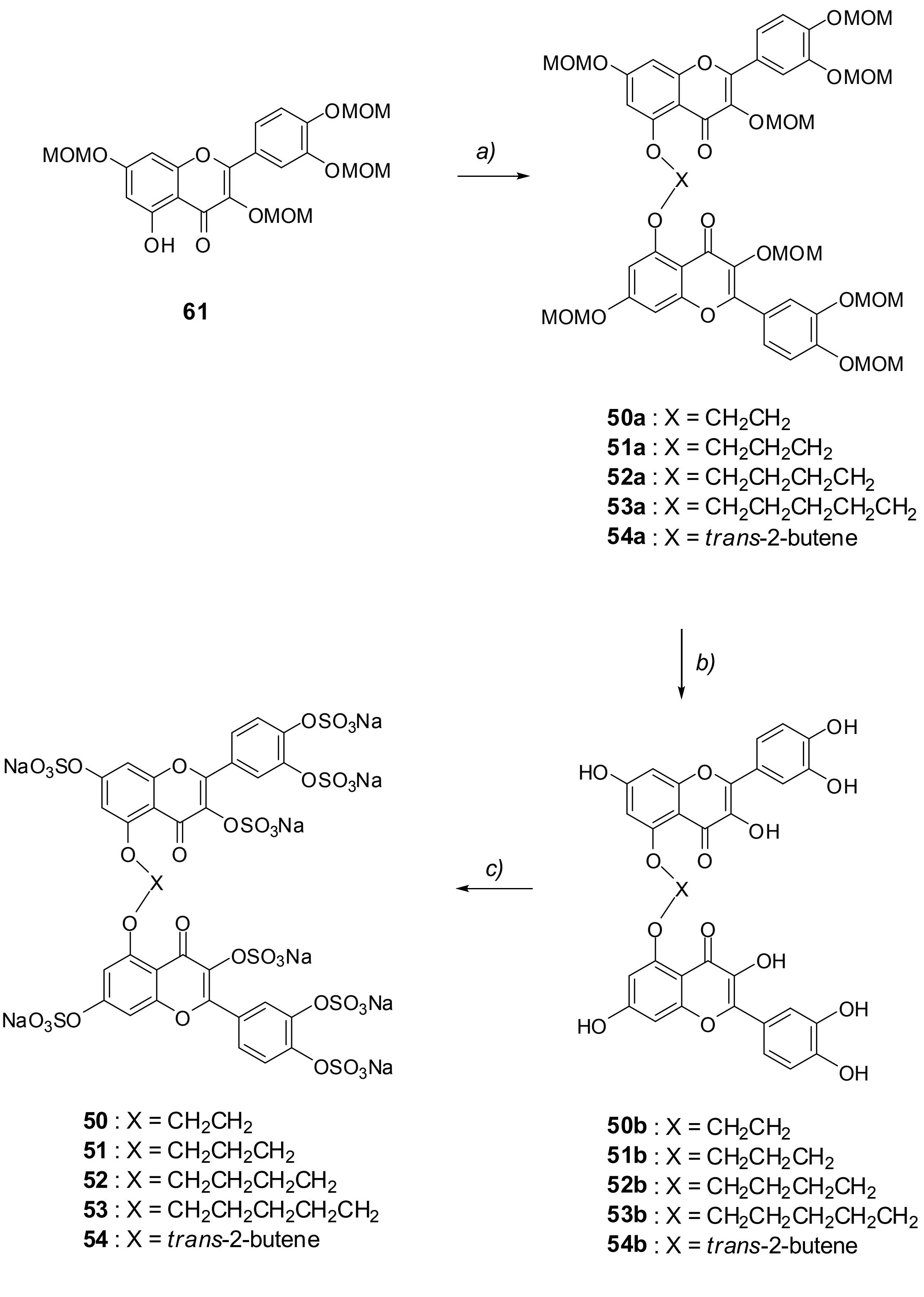

Scheme S5. *a)* K_2CO_3, dibromoalkane (0.5 equiv), DMF, rt/6 h, 85%–90%, *b) p*-Toluenesulfonic acid, MeOH, reflux/48 h, 55%–65%, *c)* SO_3:Me_3N, Et_3N, CH_3CN, microwave at 90 °C, 6 h, 75%–85%.

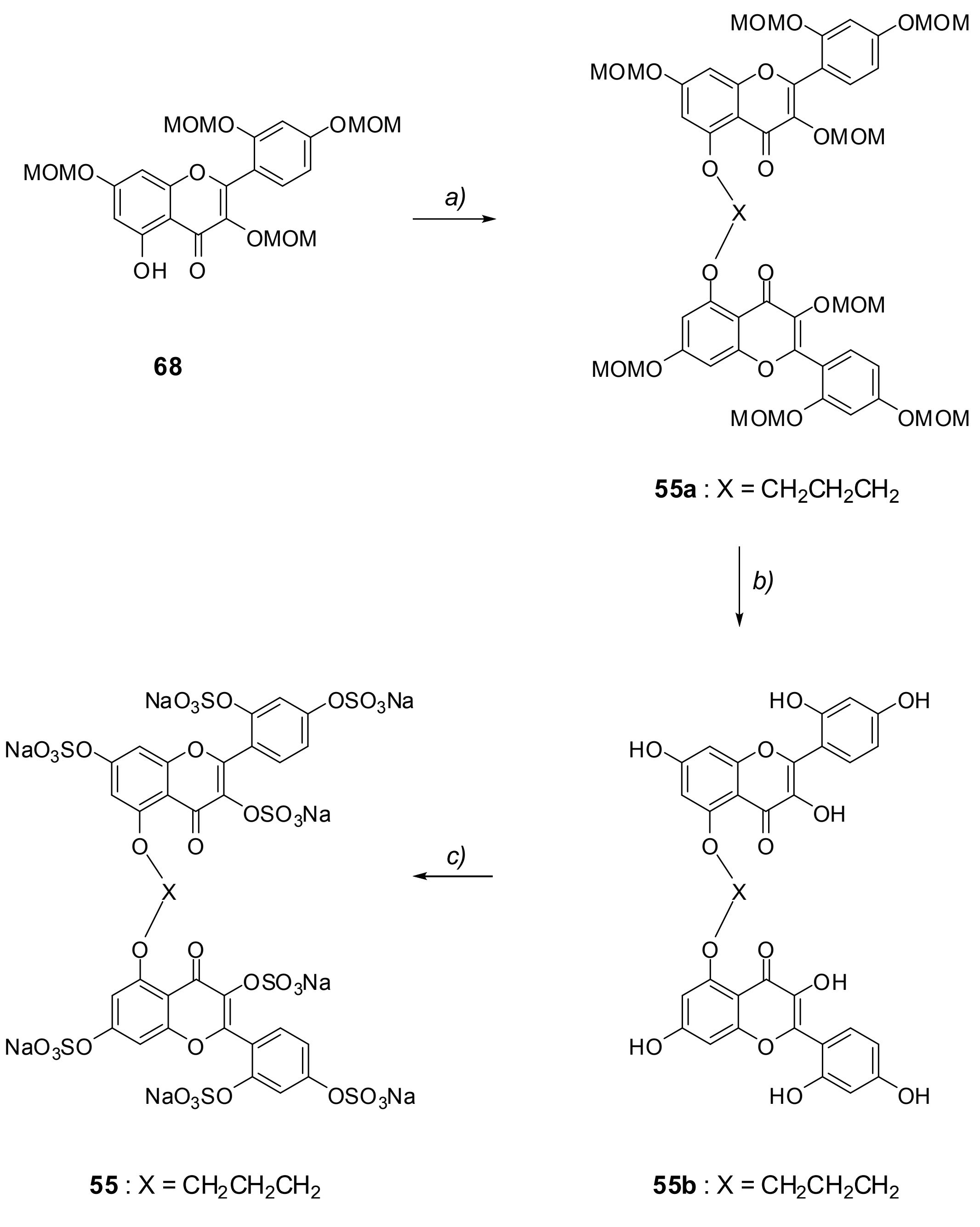

Scheme S6. *a)* K_2CO_3, dibromoalkane (0.5 equiv), DMF, rt/6 h, 85%–90%, *b) p*-Toluenesulfonic acid, MeOH, reflux/48 h, 55%–65%, *c)* SO_3:Me_3N, Et_3N, CH_3CN, microwave/6 h, 75%–85%.

General procedure for protection of flavonoid by MOM (61, 67, and 68). See above.

General procedure for flavonoid dimerization (48a–55a). To a solution of MOM-protected flavonoid (1.0 equiv) in DMF was added K_2CO_3 (2.5 equiv) and stirred for two minutes. This was followed by addition of di-bromoalkane (0.5 equiv) and stirred vigorously for 12 h. After the reaction completed as indicated from TLC the reaction mixture was diluted with EtOAC (25 mL) and water (25 mL). The organic layer was separated and the aqueous phase was extracted with EtOAc (2 × 25 mL), organic layer was washed with saturated NaCl solution (25 mL). The combined organic layers were dried over anhydrous Na_2SO_4, evaporated under reduced pressure to afford crude flavonoid dimers which were further purified using flash chromatography on silica gel (70%–85% ethyl acetate in hexanes).

(**48a**). ^{1}H-NMR ($CDCl_3$, 400 MHz): 12.32 (s, 2 H), 7.50 (d, *J* = 1.8 Hz, 2 H), 7.43 (d, *J* = 6.6 Hz, 2 H), 7.03 (d, *J* = 8.3 Hz, 2 H), 6.40 (d, *J* = 2.1 Hz, 2 H), 6.26 (d, *J* = 2.2 Hz, 2 H), 5.06 (s, 2 H), 5.03 (s, 2 H), 4.97 (s, 2 H), 4.15 (t, *J* = 4.8 Hz, 4 H), 3.298 (s, 6 H), 3.294 (s, 6 H), 3.02 (s, 6 H), 2.25–2.22 (m, 2 H). ^{13}C-NMR (DMSO-d_6, 400 MHz): 178.56, 162.98, 161.92, 156.67, 149.22, 148.71, 135.6, 128.08, 127.39, 124.46, 122.78, 116.1, 114.29, 106.61, 99.77, 97.89, 95.25, 94.21, 94.05, 65.83, 57.75, 56.41, 56.36. MS (ESI) calculated for $C_{45}H_{48}O_{20}$, $[(M+H)]^+$, *m/z* 909.27, found for $[(M+H)]^+$, *m/z* 909.231.

(**49a**). ^{1}H-NMR ($CDCl_3$, 400 MHz): 12.53 (s, 2 H), 7.71 (d, *J* = 2 Hz, 2 H), 7.63 (d, *J* = 6.6 Hz, 2 H), 7.24 (d, *J* = 8.3 Hz, 2 H), 6.62 (d, *J* = 2.2 Hz, 2 H), 6.48 (d, *J* = 2.1 Hz, 2 H), 6.23 (s, 2 H), 5.3 (s, 4 H), 5.25 (s, 4 H), 5.19 (s, 4 H), 4.77 (d, *J* = 0.8 Hz, 4 H), 3.55 (s, 6 H), 3.51 (s, 6 H), 3.25 (s, 6 H). ^{13}C-NMR (DMSO-d_6, 400 MHz): 178.54, 162.98, 161.91, 156.64, 156.49, 149.25, 148.15, 135.57, 128.76, 124.32, 122.73, 115.84, 114.48, 106.6, 99.77, 97.86, 95.26, 94.24, 94.06, 68.38, 57.75, 56.4. MS (ESI) calculated for $C_{46}H_{48}O_{20}$, $[(M+H)]^+$, *m/z* 921.27, found for $[(M+H)]^+$, *m/z* 921.241.

(**50a**). ^{1}H-NMR ($CDCl_3$, 400 MHz): 7.93 (d, *J* = 2 Hz, 2 H), 7.73 (d, *J* = 6.6 Hz, 2 H), 7.29–7.26 (m, 4 H), 6.74 (d, *J* = 8.1 Hz, 2 H), 5.33 (s, 4 H), 5.32 (s, 4 H), 5.28 (s, 4 H), 5.22 (s, 4 H), 4.64 (s, 4 H), 3.56 (s, 6 H), 3.56 (s, 6 H), 3.52 (s, 6 H), 3.25 (s, 6 H). ^{13}C-NMR (DMSO-d_6, 400 MHz): 173.60, 161.42, 159.93, 158.41, 153.33, 149.20, 146.56, 137.87, 124.94, 123.69, 117.75, 115.74, 110.46, 99.44, 97.77, 96.36, 95.70, 95.17, 94.31, 68.18, 57.58, 56.44, 56.33. MS (ESI) calculated for $C_{48}H_{54}O_{22}$, $[(M+H)]^+$, *m/z* 983.31, found for $[(M+H)]^+$, *m/z* 983.251.

(**51a**). ^{1}H-NMR ($CDCl_3$, 400 MHz): 7.88 (d, *J* = 2 Hz, 2 H), 7.66 (d, *J* = 6.6 Hz, 2 H), 7.24 (d, *J* = 8.6 Hz, 4 H), 6.63 (d, *J* = 2 Hz, 2 H), 6.53 (d, *J* = 2 Hz, 2 H), 5.29 (s, 4 H), 5.28 (s, 4 H), 5.20 (s, 4 H), 5.17 (s, 4 H), 4.45 (t, *J* = 5.4 Hz, 4 H), 3.53 (s, 6 H), 3.52 (s, 6 H), 3.46 (s, 6 H), 3.19 (s, 6 H), 2.5 (t, *J* = 5.4 Hz, 4 H). ^{13}C-NMR (DMSO-d_6, 400 MHz): 173.63, 161.43, 160.48, 158.43, 153.18, 149.09, 146.51, 137.88, 125.03, 123.65, 117.72, 115.68, 110.09, 97.92, 97.81, 95.66, 95.18, 94.25, 94.31, 65.65, 57.54, 56.37, 56.33, 56.31, 21.5. MS (ESI) calculated for $C_{49}H_{56}O_{22}$, $[(M+H)]^+$, *m/z* 997.33, found for $[(M+H)]^+$, *m/z* 997.263.

(**52a**). ^{1}H-NMR ($CDCl_3$, 400 MHz): 7.92 (d, *J* = 2.1 Hz, 2 H), 7.69 (d, *J* = 6.6 Hz, 2 H), 7.28 (d, *J* = 2.6 Hz, 2 H), 6.58 (d, *J* = 2.1 Hz, 2 H), 6.48 (d, *J* = 2.2 Hz, 2 H), 5.32 (s, 4 H), 5.32 (s, 4 H), 5.31 (s, 4 H), 5.21 (s, 4 H), 4.29 (s, 4 H), 3.57 (s, 6 H), 3.56 (s, 6 H), 3.5 (s, 6 H), 3.22 (s, 6 H), 2.27 (s, 4 H). ^{13}C-NMR (DMSO-d_6, 400 MHz): 170.64, 162.40, 159.58, 158.55, 146.54, 137.92, 125.07, 123.69, 117.78, 115.74, 105.17, 97.83, 97.59, 95.69, 95.26, 95.17, 94.29, 68.07, 57.55, 56.4, 25.32. MS (ESI) calculated for $C_{50}H_{58}O_{22}$, $[(M+H)]^+$, *m/z* 1011.34, found for $[(M+H)]^+$, *m/z* 1011.296.

(**53a**). ^{1}H-NMR ($CDCl_3$, 400 MHz): 7.91 (d, *J* = 2 Hz, 2 H), 7.71 (d, *J* = 6.6 Hz, 2 H), 7.27 (d, *J* = 3.7 Hz, 2 H), 6.68 (d, *J* = 2.2 Hz, 2 H), 6.46 (d, *J* = 2.1 Hz, 2 H), 5.31 (s, 4 H), 5.31 (s, 4 H), 5.26 (s, 4 H), 5.21 (s, 4 H), 4.15 (t, *J* = 6.5 Hz, 4 H), 3.56 (s, 6 H), 3.55 (s, 6 H), 3.52 (s, 6 H), 3.23 (s, 6 H), 2.09–2.03 (m, 4 H), 1.86–1.72 (m, 2 H). ^{13}C-NMR (DMSO-d_6, 400 MHz): 173.64, 161.35, 160.58, 158.55, 153.16, 149.12, 146.54, 137.92, 125.07, 123.69, 117.78, 115.74, 110.16, 97.83, 97.59, 95.69, 95.26, 95.17, 94.29, 69.34, 57.55, 56.4, 56.32, 28.4, 22.36. MS (ESI) calculated for $C_{51}H_{60}O_{22}$, $[(M+H)]^+$, *m/z* 1025.36, found for $[(M+H)]^+$, *m/z* 1025.251.

(**54a**). ^{1}H-NMR ($CDCl_3$, 400 MHz): 8.02 (s, 2 H), 7.92 (d, J = 2 Hz, 2 H), 7.72 (d, J = 6.6 Hz, 2 H), 7.27 (d, J = 8.7 Hz, 2 H), 6.71 (d, J = 2.1 Hz, 2 H), 6.47 (d, J = 2 Hz, 2 H), 5.31 (s, 4 H), 5.31 (s, 4 H), 5.25 (s, 4 H), 5.23 (s, 4 H), 4.72 (d, J = 3.5Hz, 4 H), 3.29 (s, 6 H), 3.29 (s, 6 H), 3.15 (s, 6 H), 3.02 (s, 6 H). ^{13}C-NMR (DMSO-d_6, 400 MHz): 173.65, 162.49, 161.35, 159.87, 158.55, 153.27, 149.17, 146.55, 137.92, 126.76, 124.97, 123.69, 117.76, 115.73, 110.21, 98.0, 97.78, 95.69, 95.59, 95.15, 94.37, 68.92, 57.58, 56.44, 56.32. MS (ESI) calculated for $C_{50}H_{56}O_{22}$, $[(M+H)]^+$, *m/z* 1009.33, found for $[(M+H)]^+$, *m/z* 1009.283.

(**55a**). ^{1}H-NMR ($CDCl_3$, 400 MHz): 7.36 (d, J = 8.5 Hz, 2 H), 6.84 (d, J = 2.2 Hz, 2 H), 6.72 (d, J = 6.3 Hz, 2 H), 6.62 (d, J = 2.1 Hz, 2 H), 6.58 (d, J = 2.2 Hz, 2 H), 5.16 (s, 2 H), 5.13 (s, 2 H), 5.10 (s, 2 H), 4.95 (s, 2 H), 4.53 (s, 4 H), 3.41 (s, 6 H), 3.40 (s, 6 H), 3.39 (s, 6 H), 2.86 (s, 6 H). ^{13}C-NMR (DMSO-d_6, 400 MHz): 173.53, 161.36, 160.11, 159.99, 159.01, 156.58, 154.32, 138.86, 132.07, 114.98, 110.95, 108.75, 103.86, 99.36, 97.50, 96.61, 94.77, 94.45, 94.35, 68.23. MS (ESI) calculated for $C_{48}H_{54}O_{22}$, $[(M+H)]^+$, *m/z* 983.31, found for $[(M+H)]^+$, *m/z* 983.281.

General procedure for MOM deprotection (48b–55b). The methoxy methyl (MOM) groups present in the flavonoid dimers were completely deprotected using following procedure. To a solution of methoxy methylated compound in methanol was taken in a flask attached to a reflux condenser and *p*-toluenesulfonic acid (catalytic) was added to it. The reaction mixture was stirred at reflux temperature, the extent of deprotection was monitored using UPLC-MS and continued until complete deprotection of all MOM groups. After completion of the reaction EtOAC (25 mL) was added to precipitate the mixture. The precipitate was filtered, washed with excess EtOAC to remove *p*-toluenesulfonic acid and dried to obtain pure polyphenolic compounds.

(**48b**). ^{1}H-NMR (DMSO-d_6, 400 MHz): 12.38 (s, 2H), 10.82-10.42 (bs, 2H), 9.8–9.2 (bs, 4H),7.73–7.61 (m, 4 H), 6.89 (d, J = 9.2 Hz, 2 H), 6.39 (d, J = 2.1 Hz, 2 H), 6.12 (d, J = 1.9 Hz, 2 H), 4.20 (t, J = 5.4 Hz, 4 H), 2.23–2.20 (m, 2H). ^{13}C-NMR (DMSO-d_6, 400 MHz): 175.83, 163.88, 160.64, 156.13, 148.95, 146.56, 146.51, 135.78, 121.99, 121.79, 115.64, 112.93, 102.99, 98.17, 93.56, 65.36, 28.85. ESI-MS calculated for $C_{33}H_{24}O_{14}$ $[(M+Na)]^+$, *m/z* 667.54, found $[(M+H)]^+$, *m/z* 645.149.

(**49b**). ^{1}H-NMR (DMSO-d_6, 400 MHz): 12.46 (bs, 2 H), 10.75 (s, 2 H), 9.77 (bs, 2 H), 9.33 (bs, 2 H), 7.85 (d, J = 2 Hz, 2 H), 7.68 (d, J = 6.3 Hz, 2 H), 6.97 (d, J = 8.7 Hz, 4 H), 6.47 (d, J = 2 Hz, 2 H), 6.25 (d, J = 2 Hz, 2 H), 4.70 (s, 4 H). ^{13}C-NMR (DMSO-d_6, 100 MHz): 175.83, 163.85, 160.64, 156.12, 149.07, 146.53, 146.18, 135.77, 128.58, 121.91, 115.76, 113.58, 103.0, 98.16, 93.57, 68.38. ESI-MS calculated for $C_{34}H_{24}O_{14}$ $[(M+Na)]^+$, *m/z* 679.55, found $[(M+H)]^+$, *m/z* 657.124.

(**50b**). ^{1}H-NMR (DMSO-d_6, 400 MHz): 10.64 (bs, 2 H), 9.7–8.2 (bs, 5 H), 7.57 (d, J = 2.2 Hz, 2 H), 7.41 (d, J = 6.2 Hz, 2 H), 6.80 (d, J = 2.4 Hz, 2 H), 6.44–6.40 (m, 4 H), 4.37 (s, 4 H). ^{13}C-NMR (DMSO-d_6, 100 MHz): 170.80, 162.33, 159.47, 157.86, 146.92, 145.03, 141.87, 137.11, 122.23, 119.08, 115.57, 114.46, 105.46, 97.55, 95.12, 67.77. ESI-MS calculated for $C_{32}H_{22}O_{14}$ $[(M+Na)]^+$, *m/z* 653.52, found $[(M+H)]^+$, *m/z* 631.197.

(**51b**). ^{1}H-NMR (DMSO-d_6, 100 MHz): 10.6 (bs, 2H), 8.4–9.0 (bs, 3H), 7.55 (d, J = 2.1 Hz, 2 H), 7.41 (d, J = 2.1 Hz, 2 H), 6.80 (d, J = 8.4 Hz, 2 H), 6.38 (d, J = 1.9 Hz, 2 H), 6.30 (d, J = 2 Hz, 2 H), 4.29 (t,

J = 5.8 Hz, 4 H), 2.26–2.22 (m, 2H). ^{13}C-NMR (DMSO-d_6, 400 MHz): 171.04, 162.36, 159.74, 157.87, 146.90, 145.01, 141.88, 137.15, 122.28, 119.09, 115.56, 114.48, 105.21, 96.32, 94.54, 64.82, 28.54. ESI-MS calculated for $C_{33}H_{24}O_{14}$ [(M+Na)]$^+$, *m/z* 667.54, found [(M+H)]$^+$, *m/z* 645.213.

(**52b**). ^{1}H-NMR (DMSO-d_6, 400 MHz): 10.58 (bs, 2 H), 9.34–8.64 (bs, 6 H), 7.57 (d, J = 2.2 Hz, 2 H), 7.42 (d, J = 6.4 Hz, 2 H), 6.80 (d, J = 8.5 Hz, 2 H), 6.37–6.32 (m, 4 H), 4.06 (s, 4 H), 2.05 (s, 4 H). ^{13}C-NMR (DMSO-d_6, 400 MHz): 170.96, 162.40, 159.90, 157.88, 146.88, 145.02, 141.72, 137.13, 122.30, 119.05, 115.56, 114.45, 105.17, 96.45, 94.41, 68.07, 25.37. ESI-MS calculated for $C_{34}H_{26}O_{14}$ [(M+Na)]$^+$, *m/z* 681.56, found [(M+H)]$^+$, *m/z* 659.261.

(**53b**). ^{1}H-NMR (DMSO-d_6, 400 MHz): 7.64 (d, J = 2.1 Hz, 2 H), 7.51–7.47 (m, 2 H), 7.13 (d, J = 7.9 Hz, 1 H), 6.88 (d, J = 8.1 Hz, 2 H), 6.46–6.37 (m, 3 H), 4.05 (t, J = 5.5 Hz, 4 H), 2.05–2.01 (m, 4 H), 1.89–1.85 (m, 2 H). ^{13}C-NMR (DMSO-d_6, 400 MHz): 170.78, 163.25, 158.89, 157.76, 146.97, 145.28, 141.82, 136.95, 122.48, 119.35, 115.86, 113.98, 105.16, 98.35, 94.35, 68.37, 25.38, 22.24. ESI-MS calculated for $C_{35}H_{28}O_{14}$ [(M+Na)]$^+$, *m/z* 695.59, found [(M+H)]$^+$, *m/z* 673.213.

(**54b**). ^{1}H-NMR (DMSO-d_6, 400 MHz): 10.73 (s, 1 H), 9.45–8.6 (bs, 6 H), 7.68 (d, J = 3.5 Hz, 2 H), 7.51–7.51 (m, 2 H), 6.90–6.87 (m, 3 H), 6.62–6.43 (m, 5 H), 4.75 (d, J = 3.5Hz, 4 H). ^{13}C-NMR (DMSO-d_6, 400 MHz): 170.89, 163.45, 158.94, 157.69, 147.78, 145.82, 142.07, 138.15, 124.67, 123.49, 122.15, 119.28, 115.58, 113.95, 105.18, 98.34, 95.47, 56.35. ESI-MS calculated for $C_{34}H_{24}O_{14}$ [(M+Na)]$^+$, *m/z* 679.55, found [(M+H)]$^+$, *m/z*657.181.

(**55b**). ^{1}H-NMR (DMSO-d_6, 400 MHz): 9.1–10.5 (bs, 5 H), 7.14 (d, J = 8.4 Hz, 2 H), 6.40 (d, J = 1.9 Hz, 2 H), 6.31 (d, J = 1.9 Hz, 4 H), 6.26 (d, J = 6.3 Hz, 2 H), 4.36 (s, 4 H). ^{13}C-NMR (DMSO-d_6, 400 MHz): 173.72, 160.81, 159.76, 156.47, 156.13, 155.68, 154.34, 137.46, 131.07, 116.34, 113.46, 112.04, 110.35, 101.83, 100.74, 68.45. ESI-MS calculated for $C_{32}H_{22}O_{14}$ [(M+Na)]$^+$, *m/z* 653.32, found [(M+H)]$^+$, *m/z* 631.197.

General procedure for chemical sulfation of *bis*-flavonoid *homo*-dimers (48–55). See above.

(**48**). ^{1}H-NMR (DMSO-d_6, 400 MHz): 12.33 (s, 2 H), 8.01 (s, 2 H), 7.64–7.56 (m, 4 H), 6.95 (d, J = 1.9 Hz, 2 H), 6.58 (d, J = 2.0 Hz, 2 H), 4.17 (t, J = 2.0 Hz, 4 H), 2.13–2.11 (m, 2 H). ^{13}C-NMR (DMSO-d_6, 100 MHz): 177.97, 160.11, 159.60, 155.32, 148.52, 145.74, 133.29, 124.47, 121.16, 119.64, 115.11, 106.08, 101.93, 97.75, 65.50, 52.77. ESI-MS calculated for $C_{33}H_{18}Na_6O_{32}S_6$ [(M+Na)]$^+$, *m/z* 1279.81, found [(M−6Na+6 HxA)+HxA]$^+$, *m/z* 1833.882.

(**49**). ^{1}H-NMR (DMSO-d_6, 400 MHz): 12.34 (s, 2H), 8.03(d, J = 2 Hz, 2 H), 7.64–7.58 (m, 4 H), 6.92 (d, J = 1.8 Hz, 2 H), 6.57 (d, J = 1.8 Hz, 2 H), 6.10 (s, 2H), 4.61 (s, 4 H). ^{13}C-NMR (DMSO-d_6, 100 MHz): 177.97, 160.14, 159.60, 156.40, 155.30, 148.29, 145.65, 133.35, 128.69, 124.35, 119.46, 114.92, 106.10, 101.96, 97.75, 68.41, 52.78. ESI-MS calculated for $C_{34}H_{18}Na_6O_{32}S_6$ [(M+Na)]$^+$, *m/z* 1291.82, found [(M−6Na+6 HxA)+HxA]$^+$, *m/z* 1845.498.

(**50**). ^{1}H-NMR (DMSO-d_6, 400 MHz): 8.19–8.09 (m, 4 H), 7.66 (d, J = 9 Hz, 2 H), 7.1 (d, J = 2 Hz, 2 H), 6.87 (d, J = 2.0 Hz, 2 H), 4.55 (s, 4 H). ^{13}C-NMR (DMSO-d_6, 100 MHz): 174.14, 158.66, 156.91,

154.16, 146.72, 142.98, 135.13, 124.6, 123.27, 119.90, 118.83, 109.70, 99.78, 68.21. ESI-MS calculated for $C_{32}H_{14}Na_8O_{38}S_8$ $[(M+Na)]^+$, *m/z* 1469.87, found $[(M-8Na+8\ HxA)+2HxA]^{2+}$, *m/z* 1142.812.

(**51**). ^{1}H-NMR (DMSO-d_6, 400 MHz): 8.07–7.99 (m, 4 H), 7.56 (d, J = 9 Hz, 2 H), 6.98 (d, J = 1.6 Hz, 2 H), 6.66 (s, 2 H), 4.2 (s, 4 H), 2.28 (s, 2 H). ^{13}C-NMR (DMSO-d_6, 100 MHz): 159.07, 157.02, 146.44, 142.92, 135.31, 124.50, 123.56, 119.81, 109.56, 66.37, 42.41. ESI-MS calculated for $C_{33}H_{16}Na_8O_{38}S_8$ $[(M+Na)]^+$, *m/z* 1483.90, found $[(M-8Na+8\ HxA)+2HxA]^{2+}$, *m/z* 1149.085.

(**52**). ^{1}H-NMR (DMSO-d_6, 400 MHz): 8.15–8.07 (m, 4 H), 7.64 (d, J = 9 Hz, 2 H), 7.04 (d, J = 1.8 Hz, 2 H), 6.72 (s, 2 H), 4.15 (s, 4 H), 2.11 (s, 4H). ^{13}C-NMR (DMSO-d_6, 100 MHz): 173.0, 159.41, 158.31, 157.01, 153.27, 146.41, 142.92, 135.30, 124.50, 123.59, 119.80, 118.77, 109.42, 100.02, 98.85, 68.81, 25.5. ESI-MS calculated for $C_{34}H_{18}Na_8O_{38}S_8$ $[(M+Na)]^+$, *m/z* 1497.95, found $[(M-8Na+8\ HxA)+2HxA]^{2+}$, *m/z* 1156.367.

(**53**). ^{1}H-NMR (DMSO-d_6, 400 MHz): 8.14–8.05 (m, 4 H), 7.63 (d, J = 9 Hz, 2 H), 7.03 (d, J = 1.8 Hz, 2 H), 6.72 (s, 2 H), 4.1 (s, 4 H), 1.94 (s, 4H), 1.74 (s, 2H). ^{13}C-NMR (DMSO-d_6, 100 MHz): 173.6, 159.21, 158.21, 157.01, 153.17, 146.21, 142.52, 135.30, 124.4, 123.95, 119.79, 118.76, 109.45, 100.32, 98.65, 67.81, 25.5, 23.8. ESI-MS calculated for $C_{35}H_{20}Na_8O_{38}S_8$ $[(M+Na)]^+$, *m/z* 1511.95, found $[(M-8Na+8\ HxA)+2HxA]^{2+}$, *m/z* 1163.163.

(**54**). ^{1}H-NMR (DMSO-d_6, 400 MHz): 8.07(d, J = 2.2 Hz, 2 H), 8.0 (d, J = 6.7 Hz, 2 H), 6.98 (d, J = 1.9 Hz, 2 H), 6.63(d, J = 1.9 Hz, 2 H), 6.39 (s, 2H), 4.64 (s, 4 H). ^{13}C-NMR (DMSO-d_6, 100 MHz): 173.24, 158.43, 158.25, 157.08, 154.28, 148.43, 143.82, 134.31, 123.50, 123.28, 119.84, 118.78, 108.45, 100.05, 98.87, 75.81. ESI-MS calculated for $C_{34}H_{16}Na_8O_{38}S_8$ $[(M+Na)]^+$, *m/z* 1495.91, found $[(M-8Na+8\ HxA)+2HxA]^{2+}$, *m/z* 1155.620.

(**55**). ^{1}H-NMR (DMSO-d_6, 400 MHz): 7.60 (d, J = 9 Hz, 2 H), 7.35 (d, J = 2 Hz, 2 H), 7.09–7.06 (m, 4 H), 6.97 (d, J = 1.8 Hz, 2 H), 4.55 (s, 4 H). ^{13}C-NMR (DMSO-d_6, 100 MHz): 174.62, 158.61, 158.46, 157.47, 156.13, 155.68, 152.14, 136.26, 131.07, 116.34, 113.46, 112.04, 110.35, 101.83, 100.74, 68.65. ESI-MS calculated for $C_{32}H_{14}Na_8O_{38}S_8$ $[(M+Na)]^+$, *m/z* 1469.87, found $[(M-8Na+8\ HxA)+2HxA]^{2+}$, *m/z* 1142.588.

3. References

1. Al-Horani, R.A.; Desai, U.R. Chemical sulfation of small molecules—Advances and challenges. *Tetrahedron* **2010**, *66*, 2907–2918.
2. Henry, B.L.; Abdel Aziz, M.; Zhou, Q.; Desai, U.R. Sulfated, low-molecular-weight lignins are potent inhibitors of plasmin, in addition to thrombin and factor Xa: Novel opportunity for controlling complex pathologies. *Thromb. Haemost.* **2010**, *103*, 507–515.
3. Karuturi, R.; Al-Horani, R.A.; Mehta, S.C.; Gailani, D.; Desai, U.R. Discovery of allosteric modulators of factor XIa by targeting hydrophobic domains adjacent to its heparin-binding site. *J. Med. Chem.* **2013**, *56*, 2415–2428.
